# Cross-seeding of WT amyloid-β with Arctic but not Italian familial mutants accelerates fibril formation in Alzheimer's disease

**DOI:** 10.1016/j.jbc.2022.102071

**Published:** 2022-05-25

**Authors:** Ruina Liang, Yao Tian, John H. Viles

**Affiliations:** School of Biological and Behavioural Sciences, Queen Mary, University of London, London, United Kingdom

**Keywords:** kinetics, co-fibrillization, amyloid, nucleation, assembly, coaggregation, AD, Alzheimer's disease, Aβ, Amyloid-β, FAD, familial AD, TEM, transmission electron microscopy, ThT, thioflavin T

## Abstract

Alzheimer’s disease (AD) involves the neurotoxic self-assembly of a 40 and 42 residue peptide, Amyloid-β (Aβ). Inherited early-onset AD can be caused by single point mutations within the Aβ sequence, including Arctic (E22G) and Italian (E22K) familial mutants. These mutations are heterozygous, resulting in an equal proportion of the WT and mutant Aβ isoform expression. It is therefore important to understand how these mixtures of Aβ isoforms interact with each other and influence the kinetics and morphology of their assembly into oligomers and fibrils. Using small amounts of nucleating fibril seeds, here, we systematically monitored the kinetics of fibril formation, comparing self-seeding with cross-seeding behavior of a range of isoform mixtures of Aβ42 and Aβ40. We confirm that Aβ40(WT) does not readily cross-seed Aβ42(WT) fibril formation. In contrast, fibril formation of Aβ40(Arctic) is hugely accelerated by Aβ42(WT) fibrils, causing an eight-fold reduction in the lag-time to fibrillization. We propose that cross-seeding between the more abundant Aβ40(Arctic) and Aβ42(WT) may be important for driving early-onset AD and will propagate fibril morphology as indicated by fibril twist periodicity. This kinetic behavior is not emulated by the Italian mutant, where minimal cross-seeding is observed. In addition, we studied the cross-seeding behavior of a C-terminal-amidated Aβ42 analog to probe the coulombic charge interplay between Glu22/Asp23/Lys28 and the C-terminal carboxylate. Overall, these studies highlight the role of cross-seeding between WT and mutant Aβ40/42 isoforms, which can impact the rate and structure of fibril assembly.

Alzheimer’s disease (AD) accounts for more than two-thirds of dementias, currently *ca*. 50 million people worldwide (1). Fundamental to the pathology of AD is the accumulation of amyloid plaques that eventually swamp the extracellular interstitium and the vasculature of the brain. The amyloid-beta peptide (Aβ) is typically 40 or 42 residues in length and is the main constituent of these amyloid deposits (2). There is now a large body of evidence to support the amyloid cascade hypothesis indicating that Aβ plays a central role in the disease (3). Levels of soluble Aβ40 tend to exceed Aβ42 in a ratio 9:1 (4, 5). However prefibrillar oligomeric assemblies of Aβ42 are thought to be the most synaptotoxic, carpeting the lipid membrane surface and forming ion channels, resulting in membrane permeability and loss of cellular homeostasis (6, 7).

A proportion (∼5%) of AD patients have inherited forms of the disease. Genetic alterations observed in early-onset familial AD (FAD) can be caused by mutations in the presenilins that are responsible for the cleavage of Aβ from the larger amyloid precursor protein, or they can arise from mutations within amyloid precursor protein itself. Some of these FADs are caused by mutations within the Aβ sequence (8, 9). Studying these mutations within the Aβ peptide should give us insights into the early processes of AD, in particular, Aβ self-association into toxic assemblies. This group of FADs, named after different regions of the world where they were first identified, are caused by single point mutations of Aβ, see Figure 1. They include Arctic (E22G), Italian (E22K), Dutch (E22Q), Iowa (D23N), and Osaka (E22Δ) type (8, 9). Interestingly, FAD mutations of Aβ are often, although not exclusively, clustered at residues 22 and 23. The *in vivo* production and degradation of Arctic, Dutch, and Italian Aβ mutants have been shown to be similar to WT Aβ (10, 11, 12). However, there is evidence to indicate that these mutations are associated with changes in the type and rate of self-association of Aβ (10, 13, 14). In particular, *in vitro* studies, using thioflavin T (ThT) as a fluorescent marker of amyloid fibrils, have indicated these Aβ mutants form amyloid more rapidly than WT Aβ, under the same conditions (10, 14, 15). It is also suggested that this enhanced fibrillogenicity reflects an increase in the proportion of toxic Aβ oligomers and protofibrils generated (11, 16). These mutations can also affect the disease phenotype, for example, Italian and Arctic mutations cause cerebral amyloid angiopathy rather than classical AD (17).

**Figure 1:**
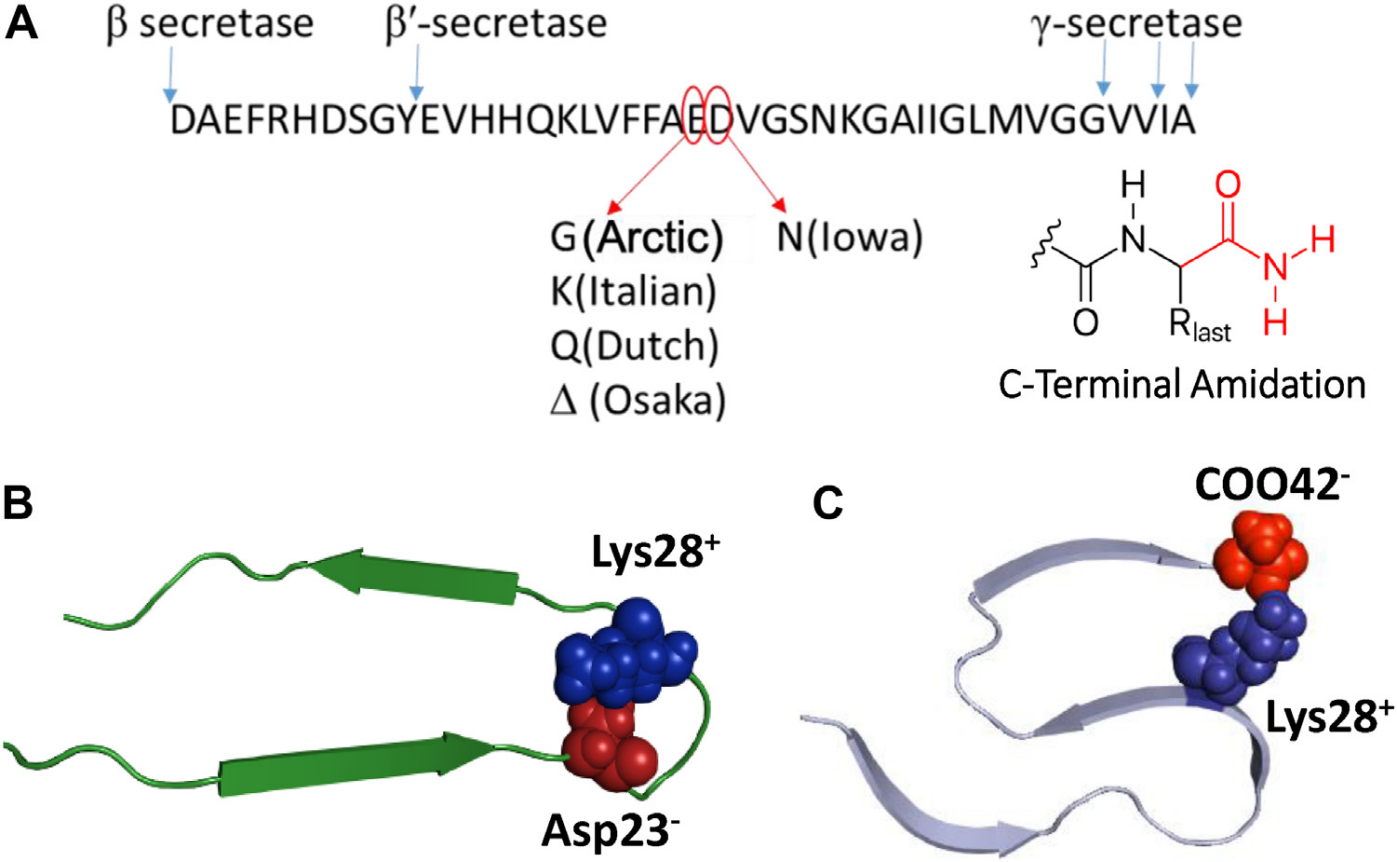
**Role of Glu22, Asp23 and the C-terminal carboxylate in Aβ40 and Aβ42 fibril struture.***A*, familial Aβ mutations at residues Glu22 and Asp23. The amyloid precursor protein (APP) cleavage sites of β- and γ-secretase are indicated by *blue arrows*. *B*, fibril topology of Aβ40 ‘U’ shaped structure (27) and (*C*) Aβ42 ‘S’ shaped structure (26). The charged salt–bridge interaction is highlighted, also shown is animation at C-terminus.

Aβ familial mutation are typically autosomal dominant, which causes heterozygote inheritance, thus people with these mutations express an equal amount of WT Aβ (9, 17, 18). WT Aβ such as Aβ40 and Aβ42 are released at synapses (in a 9:1 ratio) but also released is an equal proportion of Aβ40 and Aβ42 mutated form, in a 9:1 ratio (10, 11, 12).

It is well understood that preformed fibrils of the same Aβ isoform can self-seed or nucleate fibril formation, circumventing primary nucleation so that fibril seeds provide a surface for secondary nucleation to occur. This process dominates fibril kinetic behavior, greatly reducing the lag-phase of fibril formation (19, 20). Furthermore, these ‘parent’ seeds can propagate the same morphology in the ‘daughter’ fibril (21). There have also been numerous studies of possible cross-seeding assembly of WT Aβ40 with Aβ42 (19, 22, 23). There is now good evidence that Aβ40 and Aβ42 interact only during early oligomerization but go on to form fibrils independently and so fibrils of one isoform do not markedly impact (cross-seed) fibril formation of the other (19, 22, 23, 24), although this is not universally accepted (25). The lack of coassembly between Aβ40 with Aβ42 is thought to be due to incompatibility of their respective fibril structures, with a U-shaped topology for Aβ40 and S-shaped topology for Aβ42 fibril cores (26, 27); this results in a very different arrangement of amino acids on the surface of fibrils. This structural difference is centered on the formation of a salt-bridge (coulombic charge interaction). A salt-bridge forms in WT Aβ40 between residues Asp23 and Lys28, while in Aβ42, the salt-bridge can form between the C-terminal carboxylate of Ala42 and Lys28 (26, 27), as shown in Figure 1.

With many of the Aβ mutations that result in early-onset AD being centered at residue 22 and 23, there is a suggestion that a key feature of these mutations is their impact on the formation of a salt-bridge which influences fibril structure. The presence of equal mixtures of WT and mutant Aβ released *in vivo* raises the question- to what extent does Arctic (E22G) and Italian (E22K) mutants interact and cross-seed WT Aβ40 and Aβ42? Here, we show by systematically investigating kinetic behavior and cross-seeding studies of these Aβ mixtures that unlike Aβ40(WT), Aβ40(Arctic) can effectively cross-seed the formation of Aβ42(WT) fibrils. This is supported by propagation of the parent seed morphology to the daughter fibrils. While, Aβ40(Italian) does not markedly cross-seed with Aβ42(WT). We have further probed the impact of the salt–bridge interaction at Lys28 by studying a C-terminal amidated version of Aβ42 (Fig. 1), which lacks a C-terminal carboxylate. We show that this simple amidation is sufficient to stop seeding between WT Aβ42 and C-terminal amidated Aβ42. This indicates a vital role of the C-terminal carboxylate in influencing fibril structure.

## Results

**Figure 2:**
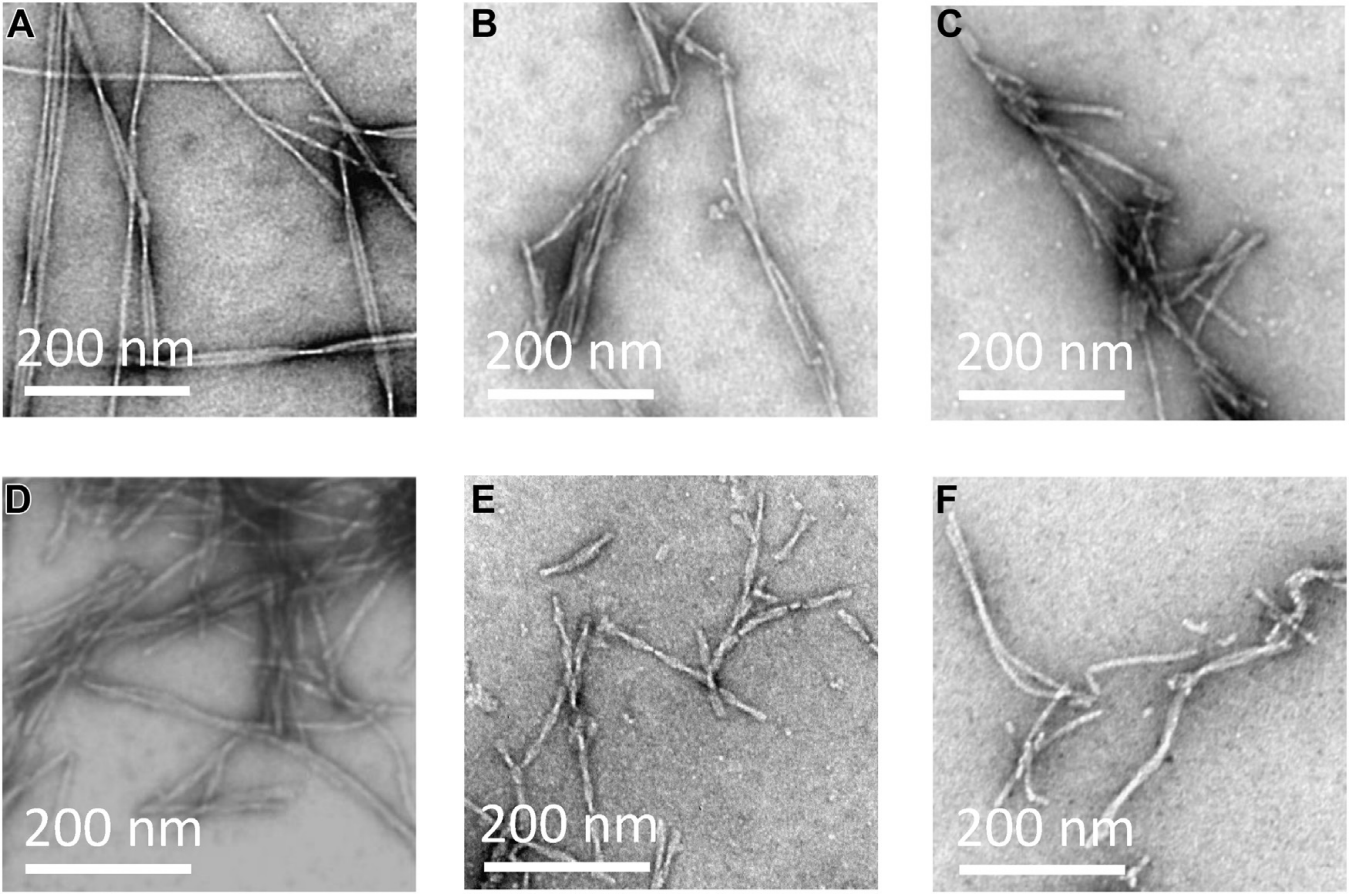
**Transmission electron microscopy images of fibrils for six Aβ isoforms.** Fibrils include: (*A*) Aβ40(WT); (*B*) Aβ40(Arctic); (*C*) Aβ40(Italian); (*D*) Aβ42(WT); (*E*) Aβ42(Arctic); (*F*) Aβ42(Italian). Negatively stained TEM images generated from 10 μM Aβ isoforms, 120 h incubation at pH 7.4, Hepes buffer (30 mM) and NaCl (160 mM). Aβ, Amyloid-β; TEM, transmission electron microscopy.

Aβ peptides were solubilized at pH 10, amyloid fibrils were then permitted to form at pH 7.4. After a number of hours, fibril assembly reaches equilibrium, as indicated by a plateau at maximal ThT fluorescence signal. Six Aβ isoforms were studied: Aβ40(WT), Aβ42(WT), Aβ40(Arctic), Aβ42(Arctic), Aβ40(Italian), and Aβ42(Italian). The amyloid fibrils generated were imaged in negative-stain by transmission electron microscopy (TEM), examples of these fibrils are shown in Figure 2. It is notable that in contrast with WT Aβ, which are typically dominated by the presence of fibrils at equilibrium, mutated isoforms also contain a number of spherical oligomers, see Fig. S1 for examples of oligomers imaged by TEM.

**Figure 3:**
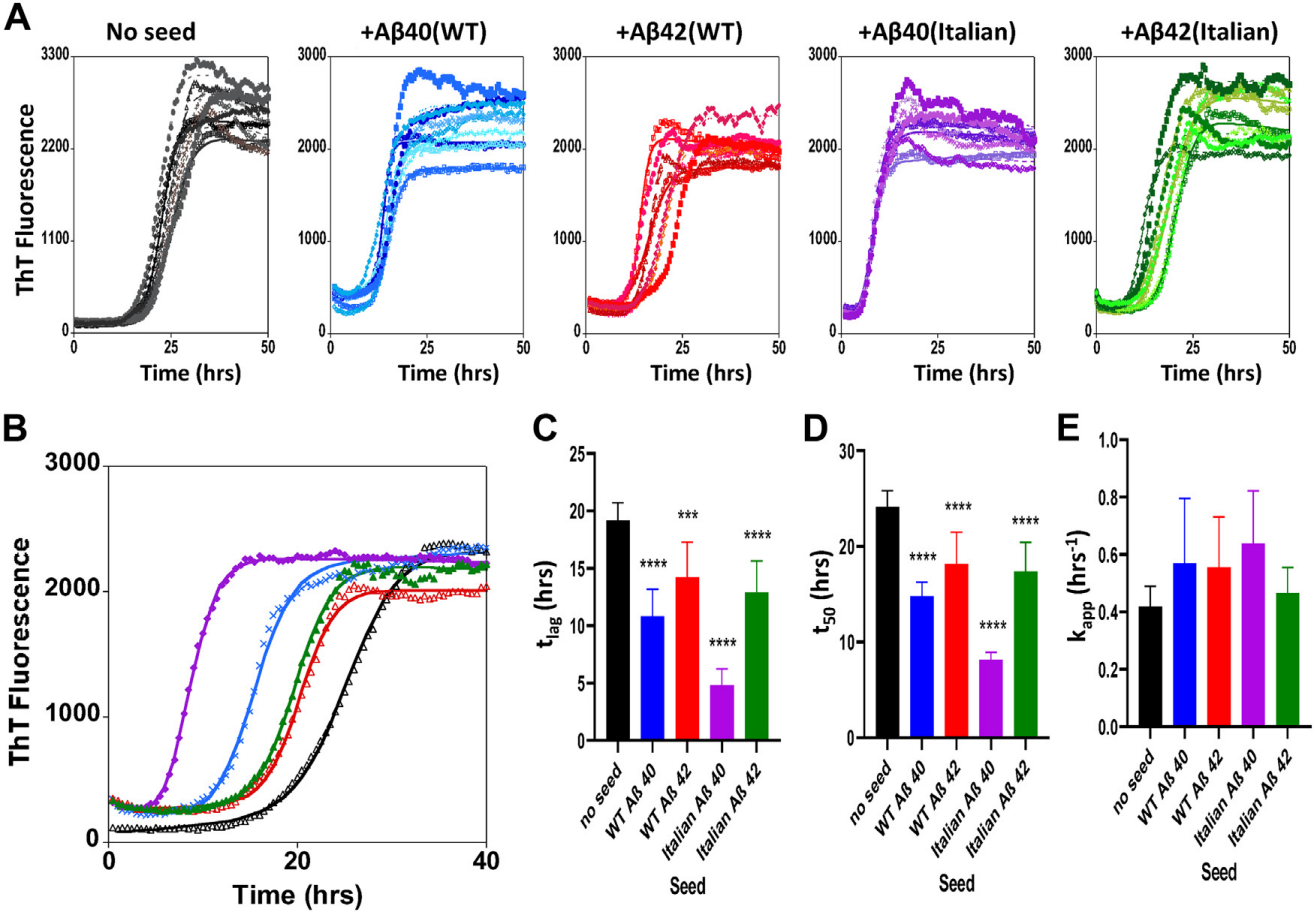
**Aβ40(Italian) monomer cross-seeding with Aβ(WT) and Aβ(Italian) fibrils**. *A*, fibril formation of monomeric Aβ40(Italian) in presence of a range Aβ isoform fibril seeds (10 % w/w): No seed (*black*); Aβ40(WT) (*blue*); Aβ42(WT) (*red*); Aβ40(Italian) (*purple*); and Aβ42(Italian) (*green*). *B*, typical representative (median) single trace of Aβ40(Italian) in the absence and presence of different seeds, same colors. Empirical kinetic parameters: *t*_*lag*_ (*C*), *t*_*50*_ (*D*), and *k*_*app*_ (*E*) of Aβ40(Italian) fibril formation, mean from n = 9 for each condition, error bars are standard deviation. Total Aβ is 10 μM at pH 7.4, Hepes buffer (30 mM) and NaCl (160 mM). Self-seeding with Aβ40(Italian) fibrils (in *purple*) most effectively nucleates fibril formation, with a large reduction in the lag-time. One-way ANOVA test, a comparison between unseeded and seeded kinetics, ∗∗∗∗*p* ≤ 0.0001, ∗∗∗*p* ≤ 0.001. Aβ, Amyloid-β.

The kinetics of fibril formation of each of these monomeric Aβ isoforms was monitored by ThT fluorescence, a fluorescent dye specific for amyloid fibrils (28). The six Aβ isoforms in their fibril form were used to seed (nucleate) the formation of fibrils from monomeric Aβ. An example of these kinetic experiments is shown in Figure 3. The kinetic formation of Aβ40(Italian) exhibits a sigmoidal growth with a lag-phase followed by a rapid elongation phase of fibril formation, culminating in an equilibrium phase where most of the monomer has formed amyloid fibrils (29, 30). The kinetic traces (in black), Figure 3*A*, shows traces for nine repeats of Aβ40(Italian) (10 μM) in the absence of a nucleating seed. This condition takes the longest to form fibrils, with a mean lag-time of 19.6 ± 0.8 h; addition of a seed 10% (1 μM) of the fibril form of Aβ40(Italian) causes a large and significant reduction in the lag-time, by half, to 9.5 ± 4.5 h (purple traces, Fig. 3). Interestingly, this nucleating effect for fibril formation is not equally significant for all of the Aβ fibril seeds. Single representative traces are overlaid in Figure 3*B* to highlight the differences. In particular, Aβ42(Italian) and Aβ42(WT) have a relatively minor seeding effect on Aβ40(Italian) monomer. Aβ40(Italian) but also Aβ40(WT) have a more substantial nucleating effect. The mean lag-times (t_lag_), t_50_, and apparent elongation rates (k_app_) are systematically compared for the five conditions in bar charts shown in Figure 3, *C*–*E*.

**Figure 4:**
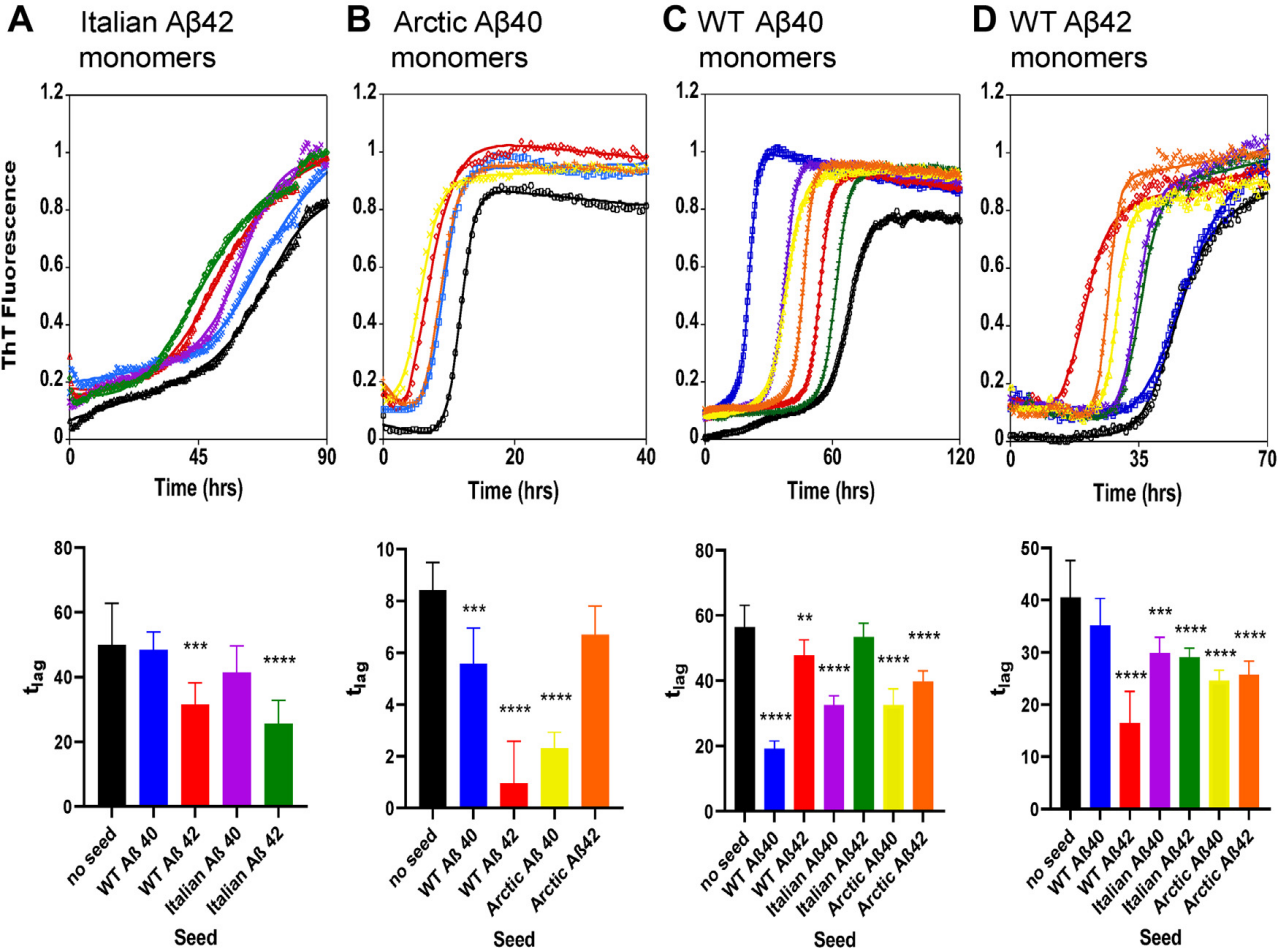
**Representative traces for coseeding and cross-seeding fibril formation and mean lag-times.** Monomers of: Aβ42(Italian) (*A*); Aβ40(Arctic) (*B*); Aβ40(WT) (*C*); and Aβ42(WT) (*D*). Representative (median) trace (*top*) and mean lag-time (*bottom*) from n = 9 for each condition, error bars are SD. Seed free is presented in *black*, cross-seeding conditions with 10% fibrils are present as: Aβ40(WT) (*blue*); Aβ42(WT) (*red*); Aβ40(Italian) (*purple*); Aβ42(Italian) (*green*); Aβ40(Arctic) (*yellow*); and Aβ42(Arctic) (*orange*). Aβ monomers were 10 μM, in Hepes (30 mM) at pH 7.4, NaCl (160 mM). ANOVA, comparison between no-seed and seeded conditions. ∗∗∗∗*p* ≤ 0.0001, ∗∗∗*p* ≤ 0.001, ∗∗*p* ≤ 0.01. See supplementary Figures for n = 9 traces for each condition. Aβ, Amyloid-β.

**Figure 5:**
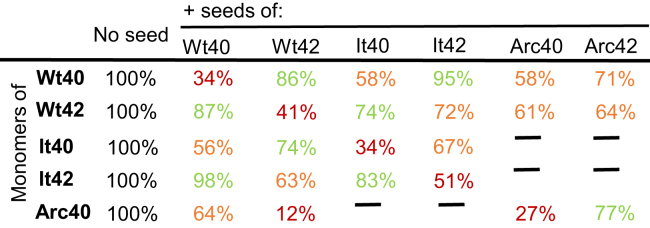
**Tabulated lag-times for cross-seeding conditions relative to nonseeded monomer**. Lag-time presented as a percentage, relative to nonseeded monomer (100%). *Red* highlighting strong seeding, *orange* indicates some seeding, and *green*, minimal seeding. WT, Italian (It), and Arctic (Arc).

Similar fibril seeding experiments have carefully been performed for monomers of Aβ40(WT), Aβ42(WT), Aβ40(Arctic), and Aβ42(Italian); this data is shown in Figs. S2–S5. A number of attempts to record Aβ42(Arctic) ThT signals were unsuccessful and so, kinetic measurements for this isoform were not possible, although it was possible to use fibrils of Aβ42(Arctic) to nucleate fibril formation. The key parameter from the data is the reduction in the lag-time and this is shown for all the isoforms studied, Figure 4. The percentage reduction in the lag-times relative to unseeded (100%) is tabulated and summarized in Figure 5; the extent of the nucleating effect has been grouped into strong seeding (red), some seeding (orange), and minimal seeding (green). In this way, Figure 5 quickly identifies which combination of Aβ monomer and seed have the strongest nucleating effects on fibril formation. Similarly, complementary t_50_ values have also been tabulated in Fig. S6. The fibril seed of the identical isoform (self-seeding) has a very strong nucleating effect, as indicated by the diagonal red highlighting in Figure 5. Furthermore, the appearance of Figure 5 exhibits a degree of mirror symmetry along the diagonal where there is the same combination of Aβ isoforms 10:90 or 90:10 mixtures of monomer and fibril. The percentage reduction in lag-time grouped in to red, orange, and green is a measure of the compatibility between the structure of the fibril seed relative to the structure of the elongating fibril. We performed independent repeat cross-seeding ThT measurements for each combination of isoforms, and the trends in the relative reduction in lag-times for specific combinations remained consistent.

As has been reported by others (19), we see from Figure 5, Aβ40(WT) fibrils do not effectively nucleate fibril formation of Aβ42(WT). Similarly, Aβ42(WT) fibrils do not markedly seed Aβ40(WT) fibril formation; lag-times are 86% and 87% relative to that of fibril formation with no seed at all (100%). In contrast, self-seeding is much more effective, with the lag-time more than halved at 34% and 41%. It is presumed that the lack of compatibility, in terms of seeding ability, between Aβ40(WT) and Aβ42(WT) is due to differences in the fundamental topology of their fibril structures (26, 27), summarized in Figure 1.

Next, we can compare how effectively the Italian and Arctic mutants of Aβ40 and Aβ42 nucleate their WT counterparts and *visa-versa*. If the basic fibril topology formed by the mutant fibrils is similar to the WT isoforms, then the seeding behavior might be expected to be similar. Indeed, to an extent, this is what we observe for the Italian mutant, in particular, Italian-Aβ40 effectively nucleates fibril formation of Aβ40(WT) with a reduction in lag-times by almost half at 56% (and 58% for the same combination in reverse). In contrast, Aβ42(Italian) has no significant seeding effect on Aβ40(WT), see Figure 5. As with WT Aβ, the behavior of cross-seeding between Aβ40(Italian) and Aβ42(Italian) is also minimal. Interestingly, the interaction of Arctic Aβ mutants with the Aβ(WT) counterparts does not follow this trend. In particular, Aβ42(WT) fibrils are able to markedly nucleate the fibril formation of Aβ40(Arctic). Despite the difference in length and sequence of these two Aβ isoforms, the nucleating ability is highly significant; indeed, nucleation is just as effective as self-seeding, with an almost eight-fold reduction of lag-times (12%), Figure 5. Similarly, in reverse, Aβ40(Arctic) fibrils will also reduce substantially lag-times of Aβ42(WT) by almost half. We note that the seeding behavior does not precisely mirror the reverse behavior, this is because the fibril surface, that causes secondary nucleation, is not the same in reverse. Strong cross-seeding in both scenarios suggests that there is a strong structural compatibility between Aβ40(Arctic) and Aβ42(WT).

The concentration dependence of seeding was also investigated for Aβ40(WT) monomer. A range of preformed fibril seed concentrations: 1%, 2%, 5%, and 10% were used, with a number of different fibril seeds: Aβ40(WT), Aβ42(WT), Aβ40(Italian), Aβ40(Arctic). Plots of t_lag_, t_50_, and k_app_ are shown *versus* log_10_ of the seed concentration, see Fig. S7. The behavior echoes the data shown in Figure 5 with almost no change in t_lag_ for Aβ40(WT) with Aβ42(WT) seeds present. In contrast, there is strong concentration dependence for self-seeding for Aβ40(WT) fibril formation. Cross-seeding with Aβ40(Italian) and Aβ40(Arctic) also exhibits almost as strong a reduction in t_lag_ with increasing preformed fibril seeds.

The slope of the kinetic curve is used to measure the empirical apparent elongation rate of fibril formation (k_app_), this parameter is strongly affected by the micro rate-constant of elongation (monomer addition on to the end of an elongating fibril) (20). The impact of adding a small amount of fibril seeds (<10%) can circumvent primary nucleation, reducing lag-times by allowing surface-catalyzed nucleation to occur but will have less of an impact on the apparent elongation rate (19, 20). As expected, in our seeded fibrillization measurements, there is little impact on k_app_ values (for example, Fig. 3*E*). Indeed, ANOVA indicates few of the seeding experiments cause a significant difference in k_app_ values; there is little variation in k_app_ with increasing seed concentrations, as shown in Fig. S7*C*.

**Figure 6:**
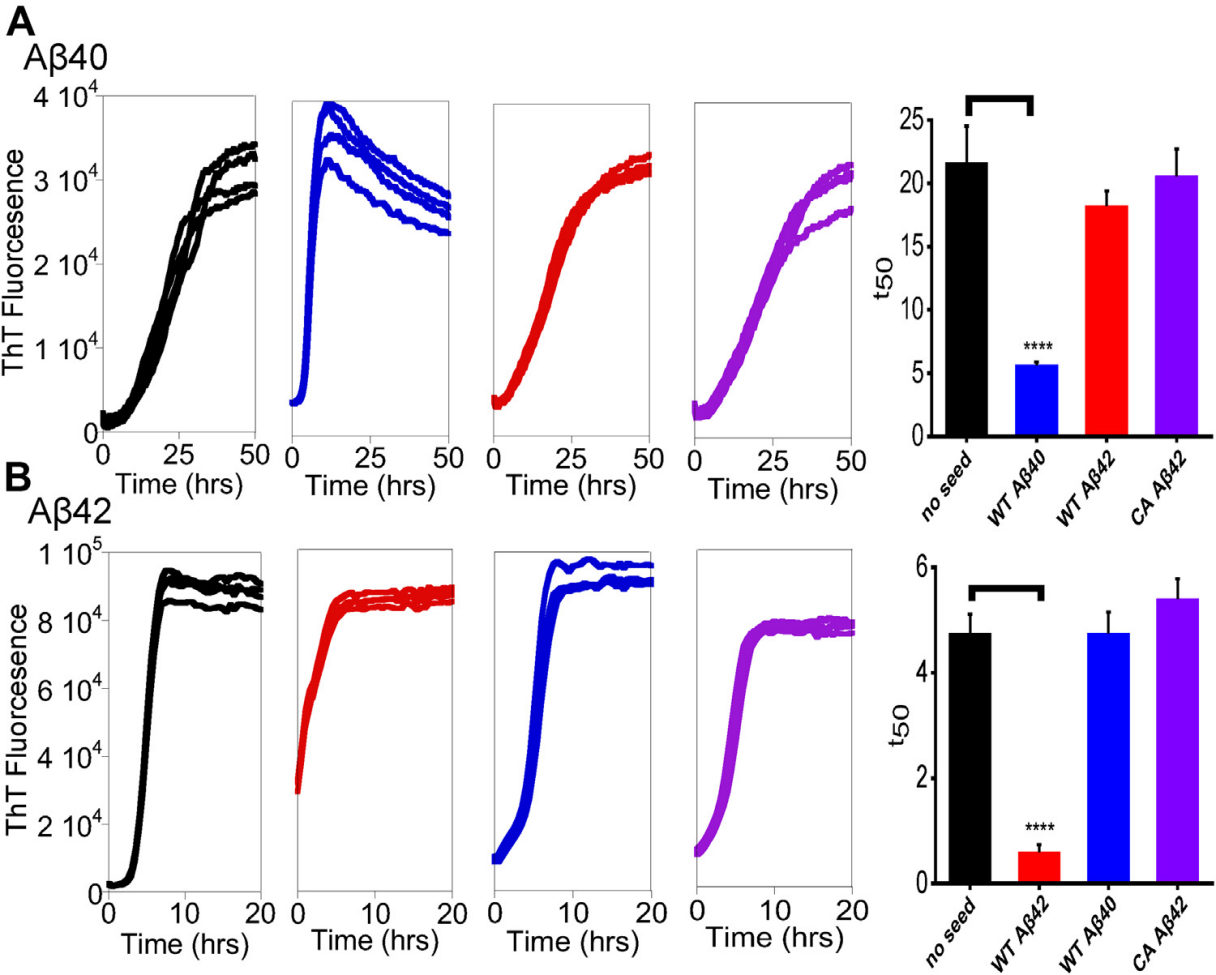
**Aβ(WT) monomer cross-seeding with Aβ(WT) and Aβ(C-Amidated) fibrils.***A*, fibril formation of monomeric Aβ40(WT) in presence of a range Aβ isoform fibril seeds (10 % w/w): no seed (*black*); Aβ40(WT) (*blue*); Aβ42(WT) (*red*); Aβ42(C-Amidated) (*purple*). The bar-charts shows *t*_*50*_ of Aβ40(WT) fibril nucleation, mean from n = 4 for each condition, compared with absence of seeding. *B*, similarly, fibril formation of monomeric Aβ42(WT) in presence of a range Aβ isoform fibril seeds (10 % w/w): no seed (*black*); Aβ42(WT) (*red*); Aβ42(WT) (*blue*); Aβ42(C-Amidated) (*purple*). Together with *t*_*50*_ of Aβ42(WT) fibril nucleation, mean from n = 4 for each condition, with and without seeding. Error bars represent standard errors of the mean (sem). One-way ANOVA test, ∗∗∗∗*p* ≤ 0.0001. Aβ, Amyloid-β.

To further probe the influence of the coulombic forces in fibril structure at Lys28 and the C-terminal carboxylate, we have investigated cross-seeding between Aβ42 and C-terminally amidated Aβ42 fibrils. This amidated Aβ42 analog readily forms amyloid fibrils as detected by ThT and imaged by negatively-stain TEM images, Fig. S8. Figure 6 shows ThT monitored fibril formation kinetics for Aβ40 and Aβ42; self-seeding is compared to the seeding with the C-terminally amidated Aβ42 isoform. Self-seeding reduces the time for nucleation considerably with the t_50_ reduced by more than half. The replacement of the negatively charged C-terminal carboxylate by the neutral amidation (Fig. 1) has a profound effect on its ability to nucleate Aβ42 fibrillization, as indicated by an unaltered lag-phase upon addition of either WT Aβ42 or Aβ40 fibril seeds, as shown in Figure 6.

**Figure 7:**
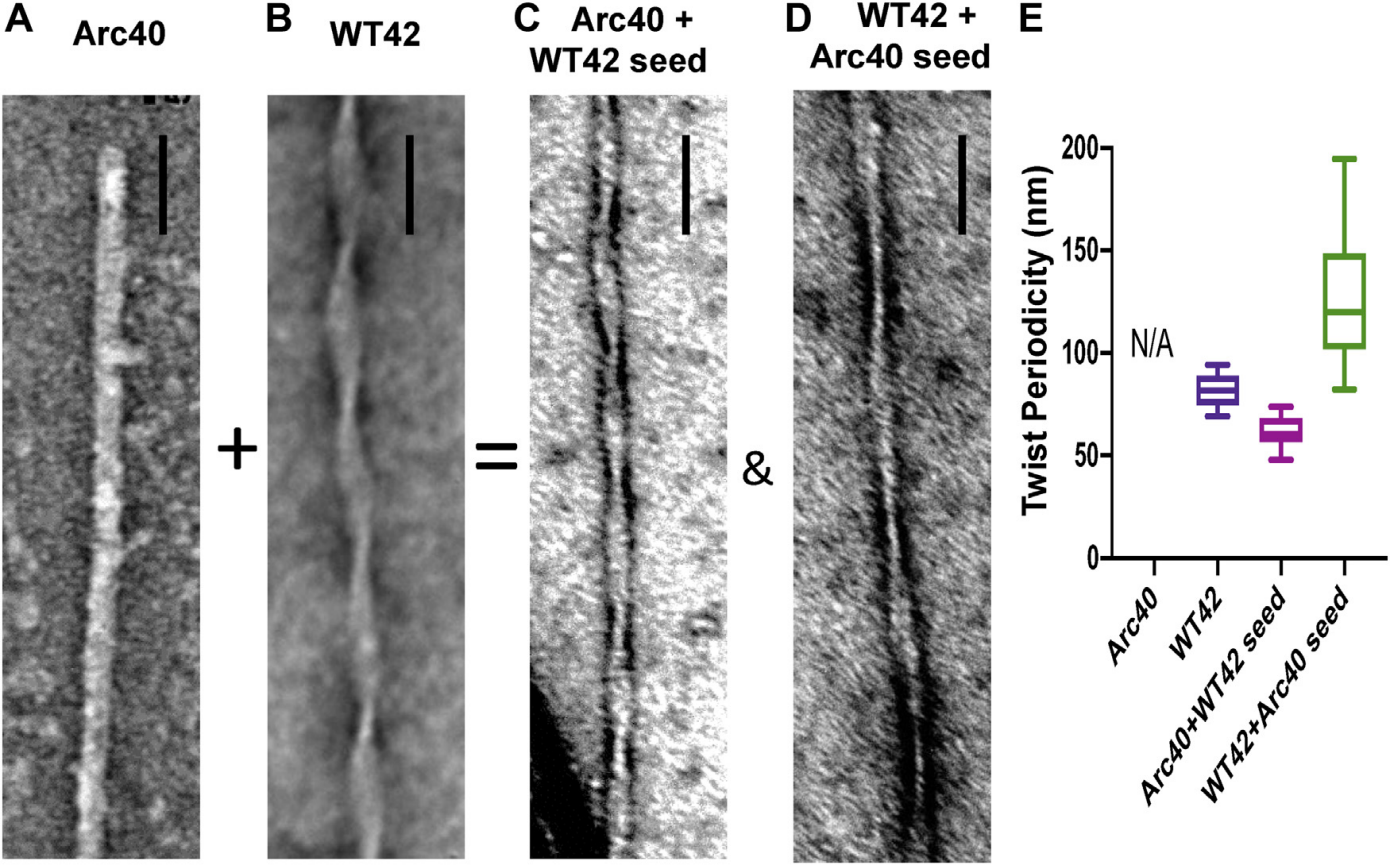
**TEM images of both seeded and unseeded Aβ42(WT) and Aβ40(Arctic).***A*, Aβ40(Arctic); (*B*) Aβ42(WT); (*C*) Aβ40(Arctic) with 10% Aβ42(WT) fibril seeds; (*D*) Aβ42(WT) with 10% Aβ40(Arctic) fibril seeds. Aβ40(Arctic) with Aβ42(WT) fibril seeds added suggest cross-seeding as the Aβ42(WT) fibril seed induces a marked twist in the otherwise untwisted Aβ40(Arctic) fibril isoform. Aβ40(Arctic) fibril seeds cause a marked extension in the periodicity of the Aβ42(WT) fibril twist from 80 nm to 125 nm. The scale bar represents 50 nm. *E*, average twist periodicity for the four conditions. Aβ, Amyloid-β; TEM, transmission electron microscopy.

Next, we wanted to determine if cross-seeding between Aβ isoforms can cause the propagation of different fibril morphologies. Inspection of fibril images by TEM shows clear differences in the extent of twist for the different Aβ isoforms. Examples of dominant representative fibrils are shown in Figures 7 and S9–S12. If there is cross-seeding between two Aβ isoforms, then you might expect the preformed fibril seed to influence the morphology of the subsequent fibrils that are formed. This parent-to-daughter seeding is well established for Aβ self-seeding (21) but may also occur by cross-seeding.

Aβ42(WT) has a pronounced repeating twist with a node-to-node periodicity of *ca* 80 nm, in contrast, Aβ40(Arctic) has an almost imperceptible twist, shown in Figure 7, *A* and *B* and S9a-b. Next, we investigated what effect cross-seeding these two isoforms would have on fibril morphology, also shown in Figures 7 and S9. We took monomeric Aβ40(Arctic) and added a small amount (10%) of fibrils of Aβ42(WT). We predicated, based on our fibril kinetic experiments, (which exhibits a substantial seeding -88% reduction in lag-times) that monomeric Aβ40(Arctic) would be impacted by Aβ42(WT) fibril seeds. As predicted, the morphology of the resulting Aβ40(Arctic) fibrils is substantially transformed; the parent seed has influenced the morphology of the daughter-fibrils. Indeed, these cross-seeded fibrils have a similar twist periodicity to parent Aβ42(WT) fibril seeds, Figure 7*E*. This transmission of fibril morphology supports the assertion that cross-seeding occurs between Aβ40(Arctic) and Aβ42(WT). The reverse cross-seeding experiment, Figure 7*D* and S9*D*, also shows substantial change in the appearance of the daughter Aβ42(WT) fibrils. The twist in the seeded fibrils although not completely lost is substantially extended with a period of *ca*. 125 nm.

The impact of other combinations of Aβ isoforms is shown in Figs. S10–S12. In these examples, the fibril seeds have little impact on subsequent fibril morphology. In particular, Aβ40(Arctic) monomer seeded with 10% of the very different fibrils from Aβ42(Arctic) or Aβ40(WT). The parent seeds have a clear twisted appearance but this morphology is not transmitted to the Aβ40(Arctic) fibrils, there is no evidence in cross-seeding, Figs. S10 and S11. Inspection of the kinetic data, Figure 5, indicated this would have been predicted with minimal reduction in lag-times of just 23% and 36%. Similarly, the dominate morphology of fibrils produced for Aβ40(WT) remains unchanged by adding 10% Aβ42(Arctic) fibril seeds, Fig. S12.

## Discussion

Most studies of the kinetics of Aβ fibril formation have been performed as single isolated Aβ isoforms, however, *in vivo* heterozygous mixture of Aβ isoforms are present at the synapse, in Arctic or Italian FAD, with a one-to-one mixture of the WT and mutant Aβ sequences present (9). We have therefore probed how mixtures of WT, with Italian or Arctic Aβ40 and Aβ42 interact with each other, by monitoring fibril growth kinetics of cross-seeding mixtures. In addition, the ability of parent seeds to confer structure onto the daughter fibril has been studied for different isoform mixtures. In this way, we have achieved a better understanding of Aβ misfolding and assembly in these inherited forms of Alzheimer’s disease, where complex mixtures of Aβ sequence and length can potentially interact together and influence Aβ assembly.

Previous studies on the assembly of mixtures of Aβ(1–40) with Aβ(1–42) show that for these two isoforms, fibrils largely form independently and exhibit biphasic primary nucleation in monomeric mixtures (19). In contrast, a truncation at the N-terminus, Aβ(11–40), also found in plaques *ex vivo* (2, 31), has been shown to readily cross-seed with Aβ(1–40) (32). It is suggested that the lack of cross-seeding between Aβ40 and Aβ42 is due to differences in the fundamental topology of the structures, forming a “U” and “S” shaped fold respectively (26, 27), see Figure 1. The topology of Aβ(11–40) and Aβ(1–40) may be similar, which allows cross-seeding to occur (32). Similarly, Aβ N-terminal extensions have also been shown to cross-seed with Aβ(1–42) (33).

Many of the familial mutations of Aβ are linked to a loss of negatively charged residues which makes Aβ more neutrally charged at pH 7.4, and this is known to increase the rate of self-association and oligomer/fibril formation (14, 34, 35). However, the familiar mutations identified are often centered at residues 22 or 23 (Fig. 1) rather than elsewhere in the Aβ sequence. This suggests that it is not simply a loss of negative charge but also a more specific structural explanation for the localization of most of the Aβ-mutations at position 22 and 23. An important aspect of the fundamental topology of WT Aβ40 is a salt-bridge formed between residues Asp23 and Lys28, while in Aβ42, the salt-bridge is formed between the C-terminal carboxylate of Ala42 and Lys28, Figure 1 (26, 27). The disruption of the salt-bridge at Asp23 in Aβ40 familial mutants is thought to be important in oligomer/fibril assembly and promoting early on-set AD (36). We have highlighted the importance of the C-terminal Ala42 carboxylate forming a coulombic interaction with Lys28 amino group by studying the impact of removal of this charge by amidation. Aβ42(Amidated) does not cross-seed with WT Aβ42, this indicates the structure of the Aβ42(Amidated) fibrils have been altered by the simple loss of the C-terminal carboxylate interaction.

The differences in cross-seeding behavior for different Aβ isoform combinations, Figure 5, is believed to be a consequence of the nature of the amyloid fibril structures each Aβ isoform is able to form. In particular, how compatible fibril structures are for the different isoforms as they cross-seed. Solid-state-NMR data of Aβ40(Arctic) suggests it contains a structure with many similarities to Aβ42(WT) fibrils with an “S” shaped topology (37, 38). This explains why cross-seeding so effectively occurs between Aβ40(Arctic) and Aβ42(WT). There is a sparsity of structural data on the Italian mutant (E22K), although FT-IR suggest anti-parallel β-sheets for Italian-Aβ42 are different from WT Aβ42 (39). In this case, there is minimal seeding with only a relatively small reduction in lag-times, (Fig. 5) which would be predicted as their fibril structures are different. Structural details of Aβ(1–40)E22Δ, the Osaka mutant (40), and the Iowa mutant Aβ(1–40)D23N (39) indicated these fibrils are structurally quite distinct from the S-shaped topology of Aβ42(WT). We therefore predict the Osaka and Iowa Aβ40 isoforms might exhibit limited cross-seeding properties with WT Aβ42, similar to the behavior of the Aβ40 Italian mutant.

Fibril morphology such as the periodicity of the twist is dependent on how protofibrils pack together (41). A point mutation will affect the arrangement of sidechains on the surface of the protofibril and so will affect fibril morphology (14). Here, we show fibril morphology can be propagating via secondary nucleation between Aβ isoforms but only when there is strong structural compatibility, such as between Aβ42(WT) and Aβ40(Arctic). This is an important observation because it indicates that *in vivo*, for these heterozygote mixtures, not only the rate of fibril formation of WT Aβ can be impacted but the Aβ mutants can also affect fibril morphology. Surface secondary nucleation is very sensitive to fibril structure with single point mutations sufficient to reduce compatibility for cofibrillization (42, 43). Our studies support the assertion that secondary nucleation on the fibril surface is sensitive to amyloid sequence but especially structure.

More widely, there is a good deal of interest in the interactions and cross-talk between very different amyloid proteins (such as Aβ and α-synuclein) which in some instances are found coaggregated *ex-vivo*, although not necessarily within the same fibril (44). Coaggregation of different amyloid proteins is likely to occur only after fibrils are formed.

Heterozygous FAD result in the release of mixtures of Aβ40/Aβ42, both WT and mutant isoforms, at the synapse (4, 5). We have shown by our cross-seeding studies that the mutant isoforms can have a profound impact on fibril formation kinetics of the different isoforms present. Cross-seeding between Aβ40(Arctic) and Aβ42(WT) is marked, with an eight-fold reduction in the lag-times (Fig. 5). Furthermore, not only the kinetics of fibril formation is implicated by heterozygote mixture but also fibril structure and morphology. This suggests that for families with inherited Arctic mutants, the abundance of Aβ40(Arctic), indeed at nine times the concentration of Aβ42(WT), is likely to act as a nucleating trigger for oligomer and fibril formation of the neuro-toxic Aβ42(WT). This is likely to contribute to the early on-set of dementia observed.

## Experimental procedures

### Aβ peptides

All Aβ peptides were purchased from EZBiolab, which were synthesized by F-moc ((*N*-(9-fluorenyl) methoxycarbonyl) chemistry. Peptides were purified with reverse-phase HPLC and then lyophilized; the sequence was confirmed with mass spectrometry. The following amino acid sequences with a free N-terminal amide and C-terminal carboxylate were generated:

Aβ40(WT): DAEFR HDSGY EVHHQ KLVFF AEDVG SNKGA IIGLM VGGVV

Aβ42(WT): DAEFR HDSGY EVHHQ KLVFF AEDVG SNKGA IIGLM VGGVVIA

Aβ40(Arctic): DAEFR HDSGY EVHHQ KLVFF AGDVG SNKGA IIGLM VGGVV

Aβ42(Arctic): DAEFR HDSGY EVHHQ KLVFF AGDVG SNKGA IIGLM VGGVV IA

Aβ40(Italian): DAEFR HDSGY EVHHQ KLVFF AKDVG SNKGA IIGLM VGGVV

Aβ42(Italian): DAEFR HDSGY EVHHQ KLVFF AKDVG SNKGA IIGLM VGGVV IA

In addition, a C-terminally amidated WT Aβ42 was also synthesized:

Aβ42(amidated): DAEFR HDSGY EVHHQ KLVFF AEDVG SNKGA IIGLM VGGVVIA_am_

### Aβ solubilization

The lyophilized Aβ peptides were solubilized in water to 0.7 mg ml^-1^ (100 μM) by adjusting to pH 10 with NaOH and left at 4 °C for 2 h. Thereafter, the Aβ solution was centrifuged at 20,000*g* at 4 °C, for 10 min. The supernatant with solubilized peptides was collected. In order to generate a seed-free preparation, the nucleating oligomeric aggregates were removed by size-exclusion chromatography, with a Superdex75 10/300 Gl column. Seed-free Aβ, termed here as monomeric, had no ThT fluorescence signal and exhibited a clear lag-phase to the nucleation polymerization reaction. The concentration of Aβ solutions were determined by measuring absorbance at 280 nm, ε_280_ = 1280 cm^-1^M^-1^, from the single tyrosine, using Hitachi U-3010 spectrophotometer. Typically, peptides contain 20% water by weight.

### Fibril growth assay

The kinetics of amyloid fibril formation were monitored with ThT, a dye which fluoresces at 487 nm upon binding to amyloid fibrils. This signal is typically proportional to the amount of amyloid fibrils present (28). Solubilized Aβ peptides were made up to 10 μM, in 160 mM NaCl and 30 mM 4-(2-hydroxyethyl)-1-piperazineethanesulfonic acid (Hepes) at pH 7.4. The kinetics of amyloid fibril formation were monitored directly after dilution to pH 7.4, by ThT binding to fibrils (20 μM ThT). The seeding experiment used 10% w/w preformed mature fibrils for each Aβ isoform (10 μM:1 μM; monomer:fibril). Multiple repeat measurements (n = 9) in a well-plate were obtained for each condition.

The samples in 96-well plate were incubated at 30 °C in fluorescence reader, BMG-Omega FLUOstar, with an excitation filter at 440 nm and emission filter at 490 nm. Flat-bottomed, polystyrene, nontissue-culture–treated plates (Falcon) were used. Fluorescence readings were taken every 30 min, with a brief 30-s gentle agitation before each reading.

### Curve fitting

The progress of Aβ assembly from monomer to fibrils follows a sigmoidal fibril growth curve, which is characterized by a lag-phase (nucleation), a growth-phase (elongation), and a plateau-phase (equilibrium). The lag-phase involves the formation of an increasing number of small nucleating assemblies, but at this stage, few fibrils are generated. The growth-phase (elongation) is dominated by the addition of Aβ monomers on to the ends of growing fibrils, which leads to rapid increases in fibril mass and ThT fluorescence (29). At equilibrium, most of the Aβ monomers have been incorporated into mature fibrils.

The fibril growth curve was fitted to the following equation (30):

$$y=\left(\upsilon_{i}+m_{i}x\right)+\frac{\left(\upsilon_{f\;}+m_{f}x\;\right)}{\left(1+e^{-\left(x\;-\;\frac{x_{0}}{\tau }\right)}\right)}$$

Empirical parameters extracted from the equation include the following: the lag-time to nucleate fibrils (t_lag_), the time at which half maximal fluorescence is reached (t_50_), and the slope of the elongation phase or the apparent elongation rate (k_app_). *y* represents fluorescent intensity, and x represents time. Initial fluorescence intensity is represented by ${\text{υ}}_{\text{i}}$, ${\text{υ}}_{\text{f}}$ represents the final fluorescence intensity maximum, and *x*_0_ is the time at which half maximal fluorescence is reached (t_50_). k_app_ = 1/τ and the lag-time (*t*_*lag*_) is taken from, t_lag_ = *x*_0_ - 2τ (30).

Empirical kinetic parameters are presented as means of nine traces, all error bars shown are for SD (σ). Note with n = 9 kinetic traces, the 95% confidence interval in these error bars is: 2.3(σ/√9) = 0.77σ. A one-way ANOVA test was used to measure the significance in the difference between seeded with nonseeded kinetic parameters (*p* values).

### Transmission electron microscopy

Aβ fibril samples were prepared with the same protocol for Aβ fibril growth assay but without ThT addition. The Aβ fibrils were collected after the fluorescence level reach a maximum, in adjacent wells. Aβ fibrils sample were added onto glow-discharged carbon-coated copper grids, using the Pelco EasiGlow glow discharge unit. Grids were negatively stained with uranyl acetate (2 % w/v), using the droplet method, with water washes before addition of stain. Images were recorded by a JEOL JEM1230 or a JEM2100 electron microscope. Node-to-node fibril periodicity was measured with image J.

## Data availability

All data is contained within this article.

## Supporting information

This article contains supporting information. Supplemental Figures S1–S12.

## Conflict of interest

The authors declare that they have no conflicts of interest with the contents of this article.
