# Supplementary material for: Cross-seeding of WT amyloid-β with Arctic but not Italian familial mutants accelerates fibril formation in Alzheimer's disease
Source: J Biol Chem. 2022 May 25;298(7):102071. doi: 10.1016/j.jbc.2022.102071 (PMC9243174; doi:10.1016/j.jbc.2022.102071)
Supplement: Supplemental Figures S1–S12 [file mmc1.pdf]

Supplemental:

Cross-seeding of wild-type Amyloid-β with Arctic but not Italian familial mutants accelerates fibril formation in Alzheimer's Disease

Ruina Liang , Yao Tian and John H. Viles

Supplemental Figures S1 –S12

Supplemental Figure S1: TEM images at equilibrium phase Arctic and Italian Isoforms.

Supplemental Figure S2: Aβ42(Italian) monomer cross-seeding with Aβ(WideType) and Aβ(Italian) fibrils.

Supplemental Figure S3: Aβ40(Arctic) monomer cross-seeding with Aβ(WideType) and Aβ(Arctic) fibrils.

Supplemental Figure S4: Aβ40(WildType) monomer cross-seeding with Aβ(WildType), Aβ(Italian) and Aβ(Arctic) fibrils.

Supplemental Figure S5: Aβ42(WildType) monomer cross-seeding with Aβ(Arctic), Aβ42(Italian) and Aβ(WildType) fibrils.

Supplemental Figure S6: Tabulation of mean half-times for cross-seeding conditions.

Supplemental Figure S7: Cross-seeding of WildType Aβ40 fibrillization with seeds at different concentrations.

Supplemental Figure S8: C-Amidated Aβ42

Supplemental Figure S9: TEM images of both seeded and unseeded Aβ42(WildType) and Aβ40(Arctic).

Supplemental Figure S10: TEM images of both seeded and unseeded Aβ40(Arctic).

Supplemental Figure S11: TEM images of both seeded and unseeded Aβ40(Arctic).

Supplemental Figure S12: TEM images of both seeded and unseeded Aβ40(WildType).

a) Aβ40(Arctic)

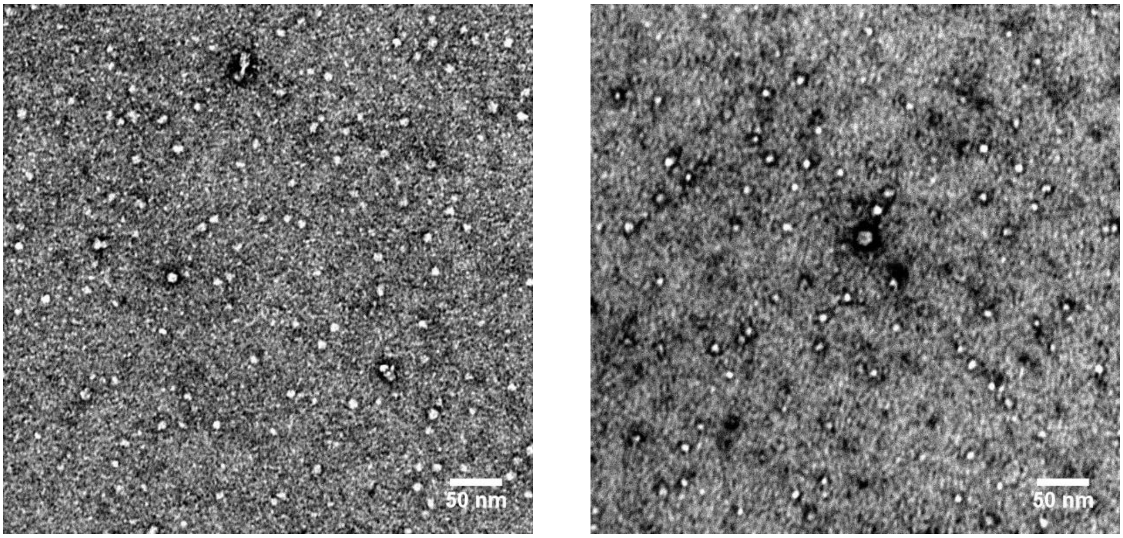

b) Aβ42(Arctic)

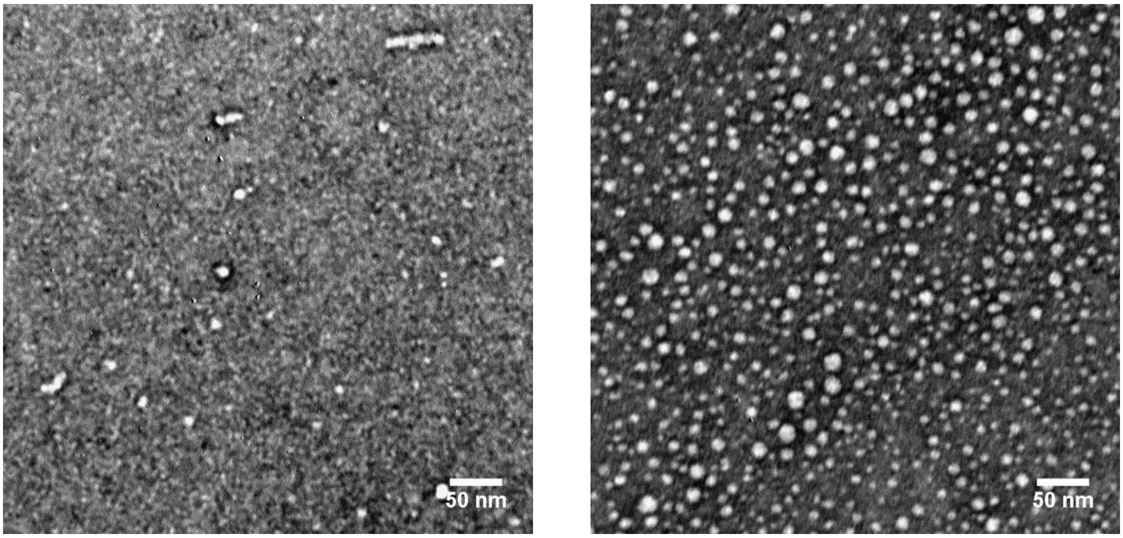

Supplemental Figure S1: TEM images at equilibrium phase Arctic and Italian Isoforms. Many oligomers as well as fibrils are observed at equilibrium for four mutant Aβ isoforms: a) Aβ40(Arctic); b) Aβ42(Arctic); c) Aβ40(Italian); d) Aβ42(Italian). Negatively stained TEM images generated from 10 µM Aβ isoforms. Once ThT has reached maximum after 50 hrs incubation at pH 7.4, HEPES buffer (30 mM) and NaCl (160 mM).

c) Aβ40(Italian)

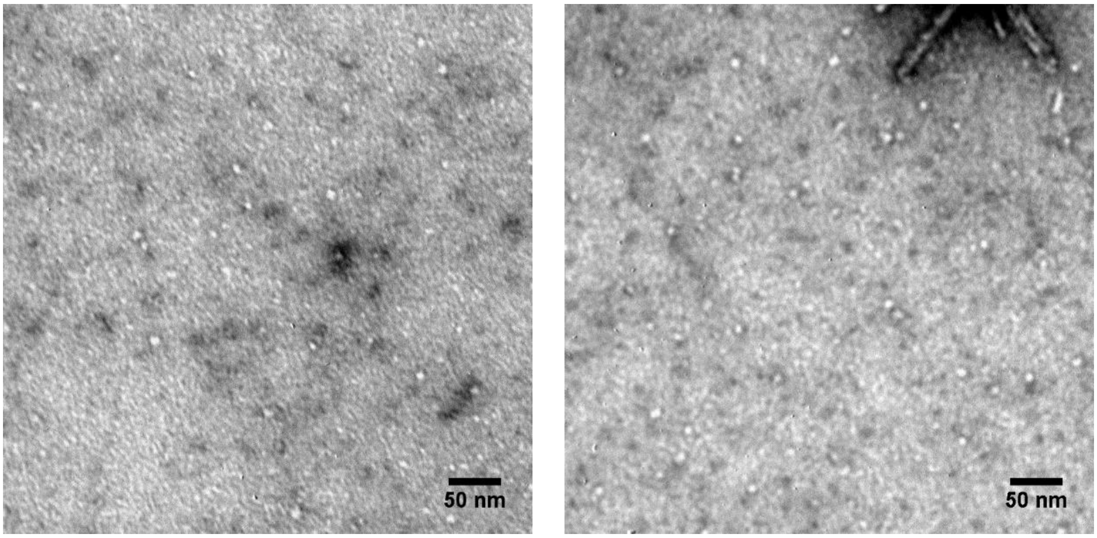

d) Aβ42(Italian)

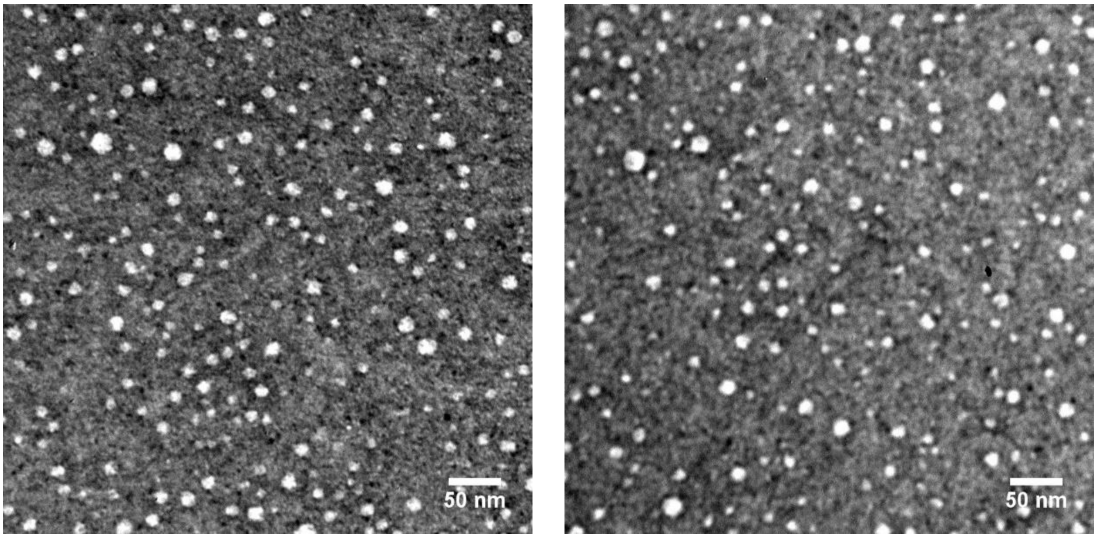

Supplemental Figure S1: TEM images at equilibrium phase Arctic and Italian Isoforms. Many oligomers as well as fibrils are observed at equilibrium for four mutant Aβ isoforms: a) Aβ40(Arctic); b) Aβ42(Arctic); c) Aβ40(Italian); d) Aβ42(Italian). Negatively stained TEM images generated from 10 µM Aβ isoforms. Once ThT has reached maximum after 50 hrs incubation at pH 7.4, HEPES buffer (30 mM) and NaCl (160 mM).

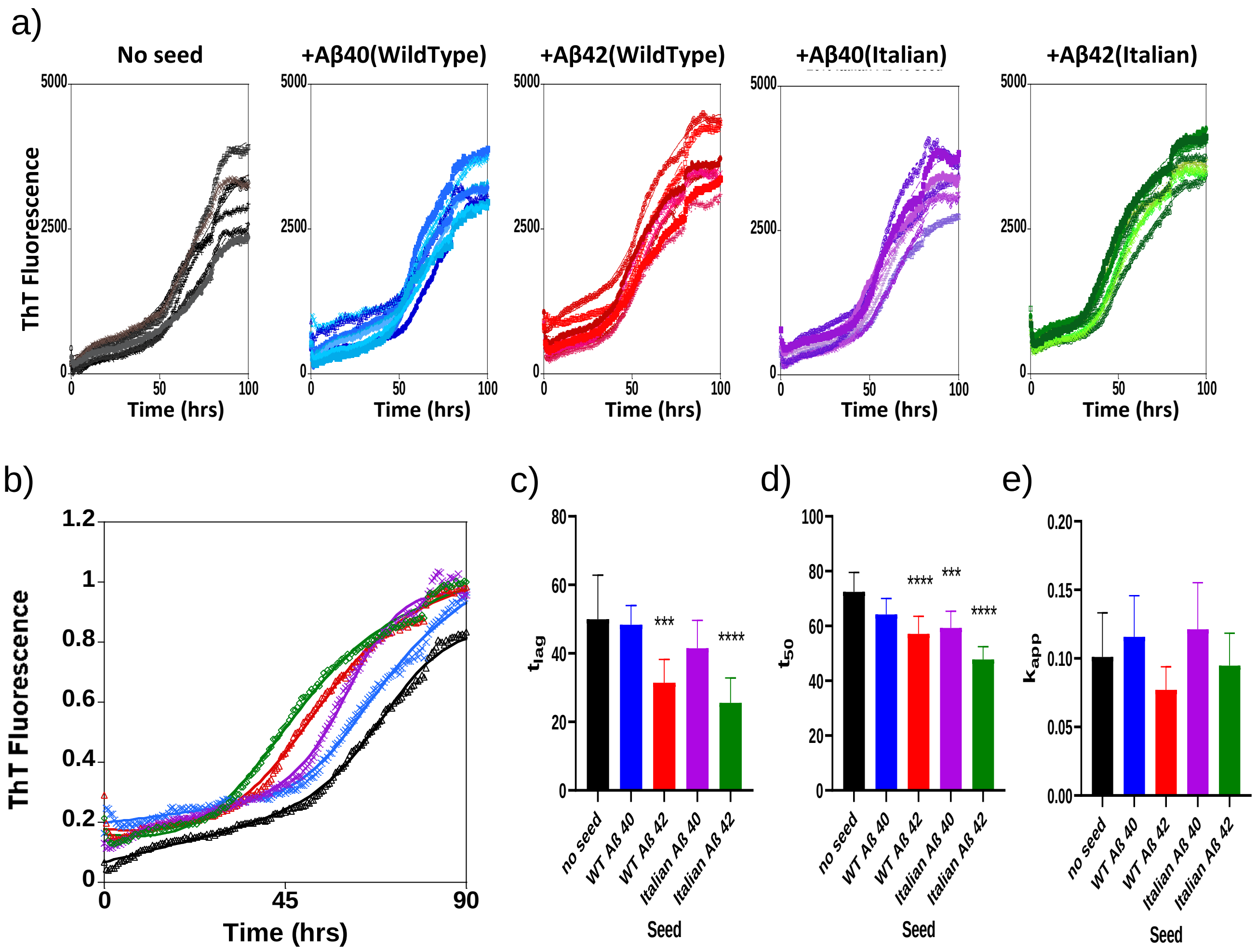

Supplemental Figure S2: Aβ42(Italian) monomer cross-seeding with Aβ(WideType) and Aβ(Italian) fibrils. a) Fibril formation of monomeric Aβ42(Italian) in presence of a range Aβ isoform fibril seeds (10 % w/w): No seed (black); Aβ40(WildType) (blue); Aβ42(WildType) (red); Aβ40(Italian) (purple) and; Aβ42(Italian) (green). b) Typical representative (median) single trace of Aβ42(Italian) in the absence and presence of different seeds, same colours. Empirical kinetic parameters for: t_{lag} (c); t_{50} (d) and k_{app} (e) of Aβ42(Italian) fibril formation, mean from n=9 for each condition, error bars are standard deviation. Total Aβ is 10 μM at pH 7.4. Self-seeding with Aβ42(Italian) fibrils (in green) most effectively nucleates fibril formation, with a large reduction in the lag-time. One-way ANOVA test, a comparison between unseeded and seeded kinetics ****P ≤ 0.0001, ***P ≤ 0.001.

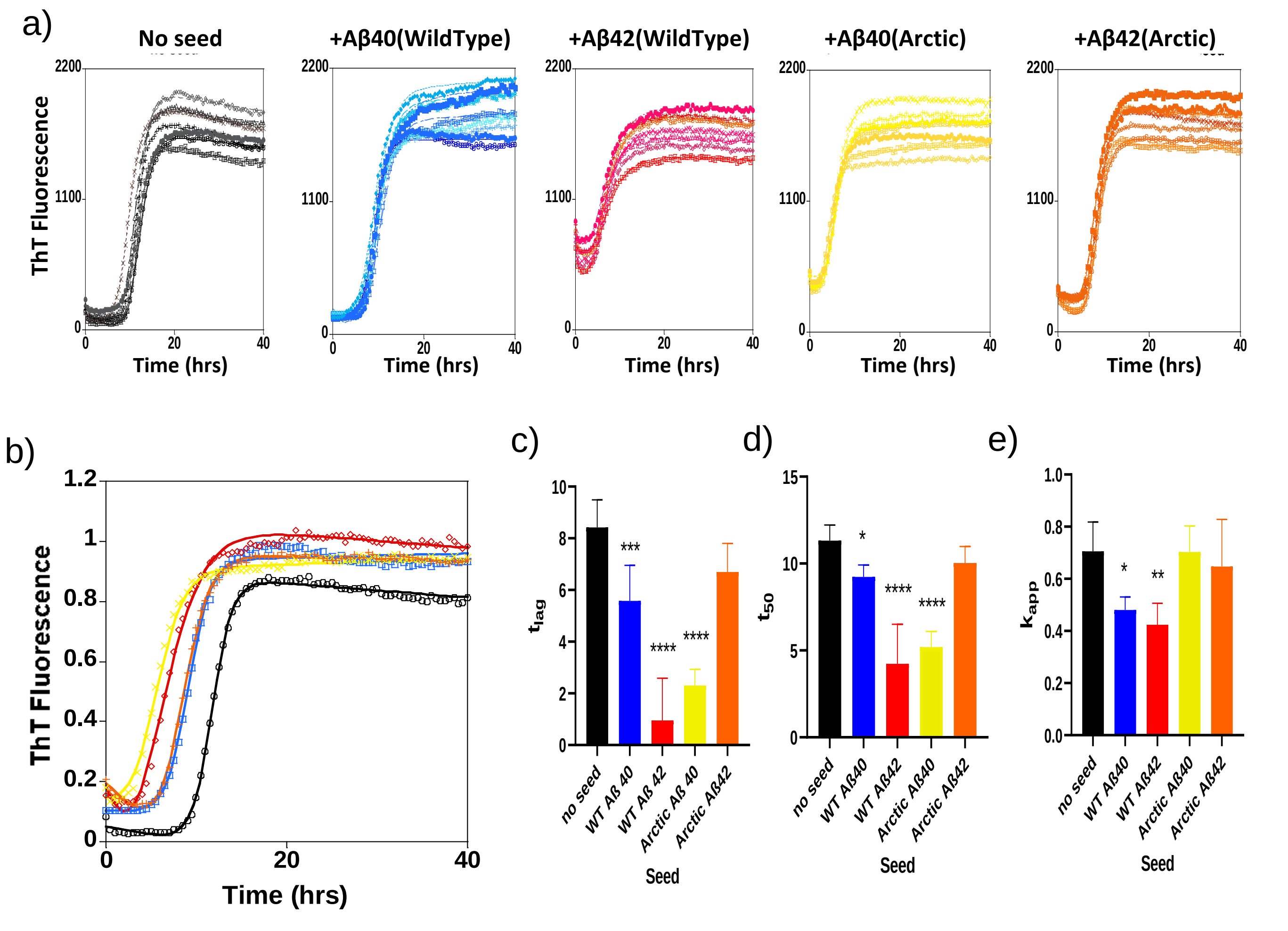

Supplemental Figure S3: Aβ40(Arctic) monomer cross-seeding with Aβ(WideType) and Aβ(Arctic) fibrils. a) Fibril formation of monomeric Aβ40(Arctic) in presence of a range Aβ isoform fibril seeds (10 % w/w): No seed (black); Aβ40(WildType) (blue); Aβ42(WildType) (red); Aβ40(Arctic) (yellow) and; Aβ42(Italian) (orange). b) Typical representative (median) single trace of Aβ40(Arctic) in the absence and presence of different seeds, same colours. Empirical kinetic parameters: t_{lag} (c), t_{50} (d) and k_{app} (e) of Aβ40(Arctic) fibril formation, mean from n=9 for each condition, error bars are standard deviation. Total Aβ is 10 μM at pH 7.4. Self-seeding with Aβ40(Arctic) fibrils (in yellow) effectively nucleates fibril formation, surprisingly Aβ42(WildType) (in red) is also very effective at nucleating fibril formation, with a large reduction in the lag-time. One-way ANOVA test, a comparison between unseeded and seeded kinetics , *P ≤ 0.05, **P ≤ 0.01, ***P ≤ 0.001, ****P ≤ 0.0001.

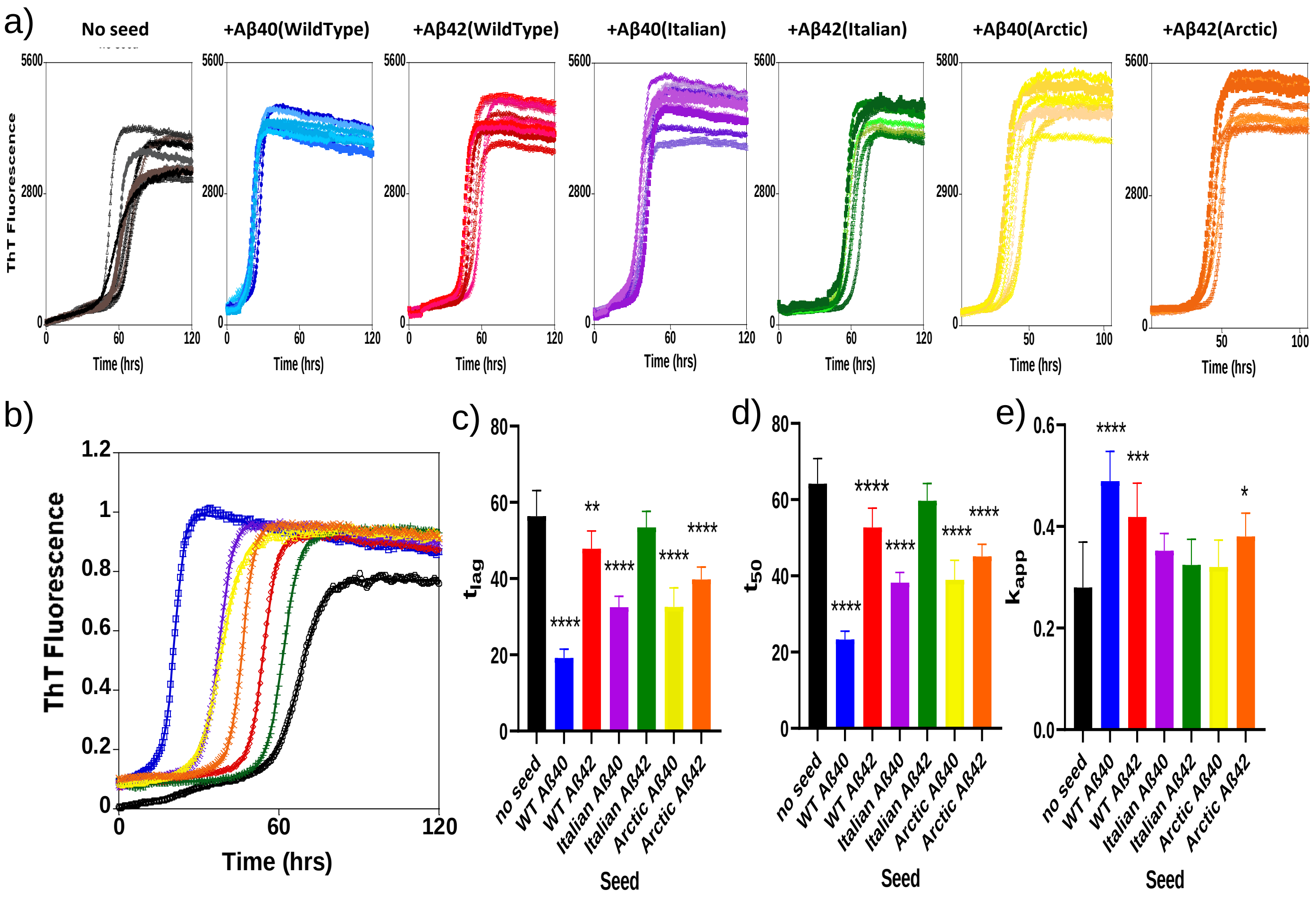

Supplemental Figure S4: Aβ40(WildType) monomer cross-seeding with Aβ(WildType), Aβ(Italian) and Aβ(Arctic) fibrils. a) Fibril formation of monomeric Aβ40(WildType) in presence of a range Aβ isoform fibril seeds (10 % w/w): no seed (black); Aβ40(WildType) (blue); Aβ42(WildType) (red); Aβ40(Italian) (purple); Aβ42(Italian) (green); Aβ40(Arctic) (yellow) and; Aβ42(Arctic) (orange); b) Typical representative (median) single trace of Aβ40(WildType) in the absence and presence of different seeds. Empirical kinetic parameters: t_{lag} (c), t_{50} (d) and k_{app} (e) of Aβ40(WildType) fibril nucleation, mean from n=9 for each condition, error bars are standard deviation. Total Aβ is 10 μM at pH 7.4. Self-seeding with Aβ40(WildType) fibrils (in blue) effectively nucleates fibril formation, with a large reduction in the lag-time. One-way ANOVA test, a comparison between unseeded and seeded kinetics: $*P \leq 0.05$, $**P \leq 0.01$, $***P \leq 0.001$, $****P \leq 0.0001$.

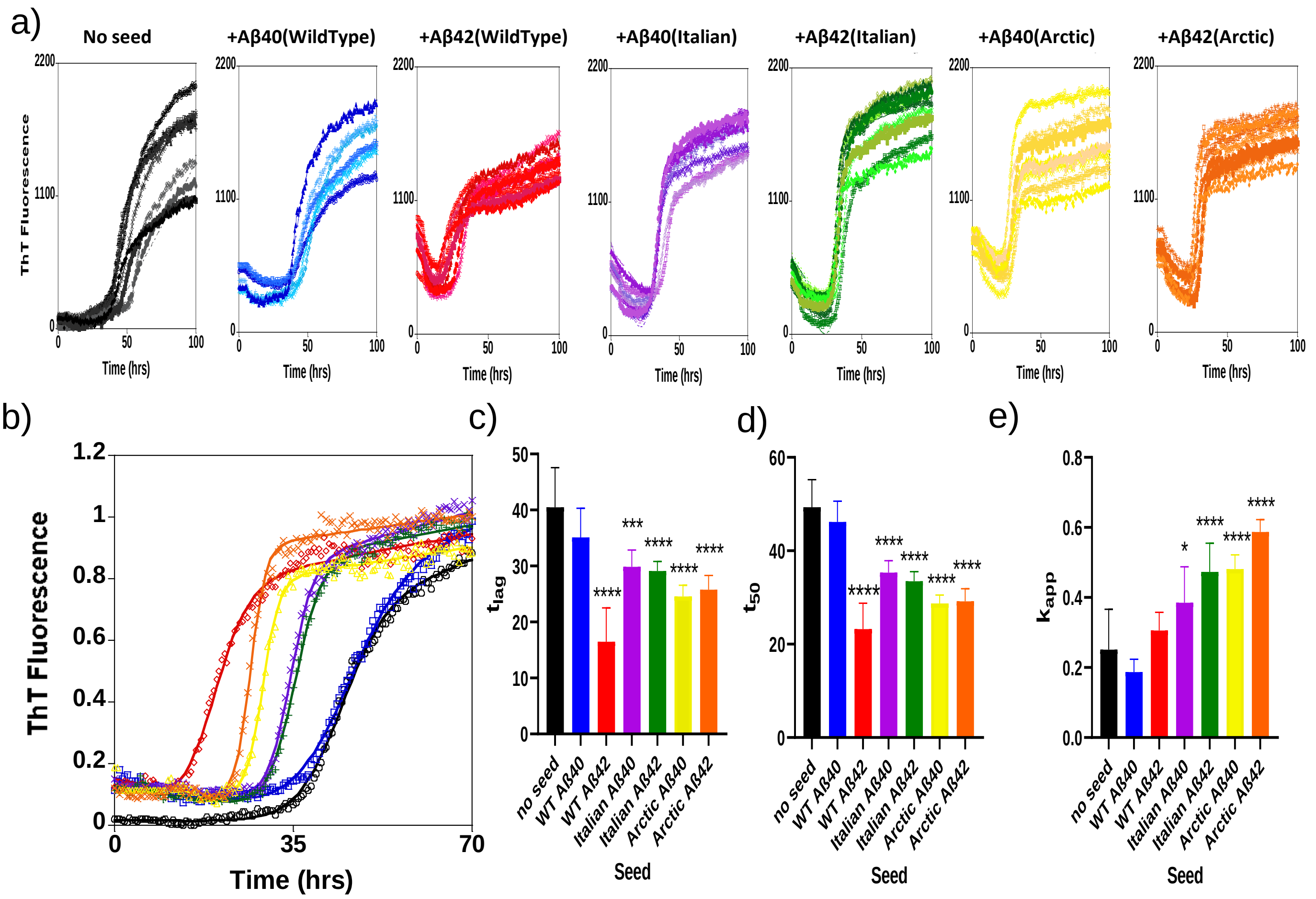

Supplemental Figure S5: Aβ42(WildType) monomer cross-seeding with Aβ(Arctic), Aβ42(Italian) and Aβ(WildType) fibrils. a) Fibril formation of monomeric Aβ42(WildType) in presence of a range Aβ isoform fibril seeds (10 % w/w): No seed (black); Aβ40(WildType) (blue); Aβ42(WildType) (red); Aβ40(Italian) (purple); Aβ42(Italian) (green); Aβ40(Arctic) (yellow) and; Aβ42(Italian) (orange). b) Typical representative (median) single trace of Aβ42(WildType) in the absence and presence of different seeds, same colours. Empirical kinetic parameters: t_{lag} (c), t_{50} (d) and k_{app} (e) of Aβ42(WildType) fibril formation, mean from n=9 for each condition, error bars are standard deviation. Total Aβ is 10 μM at pH 7.4. Self-seeding with Aβ42(WildType) fibrils (in red) effectively nucleates fibril formation as do both Arctic mutants, with a large reduction in the lag-time. One-way ANOVA test, a comparison between unseeded and seeded kinetics $*P \leq 0.05$, $***P \leq 0.001$, $****P \leq 0.0001$.

t_{50}(%)		No seed	+ seeds of: Wt40	Wt42	It40	It42	Arc40	Arc42
Monomers of	**Wt40**	100%	36%	82%	60%	93%	61%	70%
	**Wt42**	100%	93%	47%	72%	68%	58%	59%
	**It40**	100%	61%	79%	34%	72%	—	—
	**It42**	100%	87%	79%	82%	66%	—	—
	**Arc40**	100%	82%	37%	—	—	46%	87%

Supplemental Figure S6: Tabulation of mean half-times for cross-seeding conditions. a) summary of half-time values for cross-seeding conditions in hours b) summary of half-times for cross-seeding conditions relative to non-seeded monomer. $\mathbf{t}_{50}$ presented as a percentage, relative to non-seeded monomer (100%). Red highlighting strong seeding, orange indicates some seeding, and green, minimal seeding. Wild-Type (Wt), Italian (It) and Arctic (Arc).

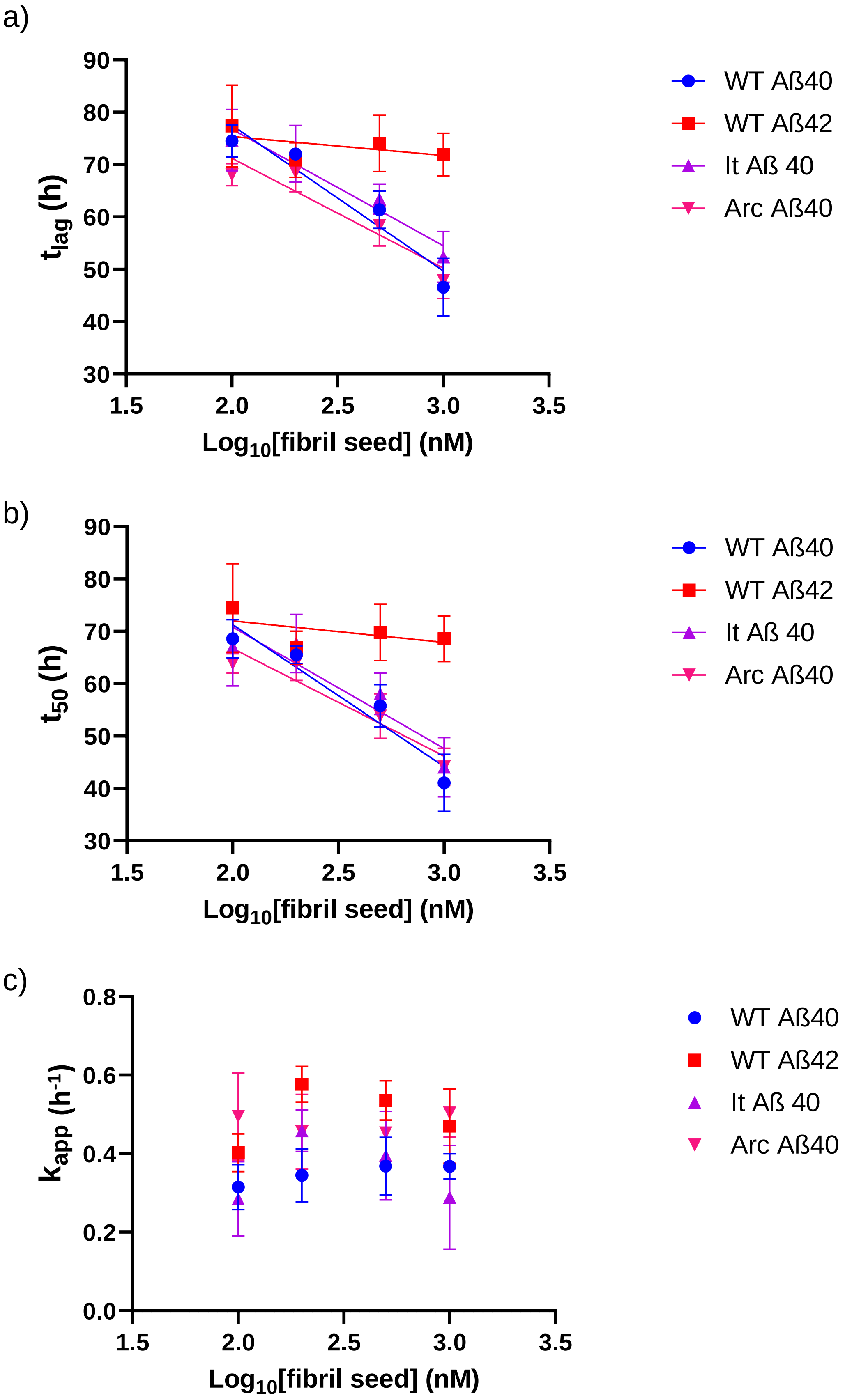

Supplemental Figure S7: Cross-seeding of WildType Aβ40 fibrillization with seeds at different concentrations. a,b,c) Lag time t_{lag}(a), half t_{50} (b)and k_{app} of WildType Aβ40 aggregation in the of fibril seeds: blue, WildType Aβ40; red, WildType Aβ42; purple, Italian Aβ40; and magenta, Arctic Aβ40. Symbols and error bars are the average and standard deviation of the four cross-seeding experiments.

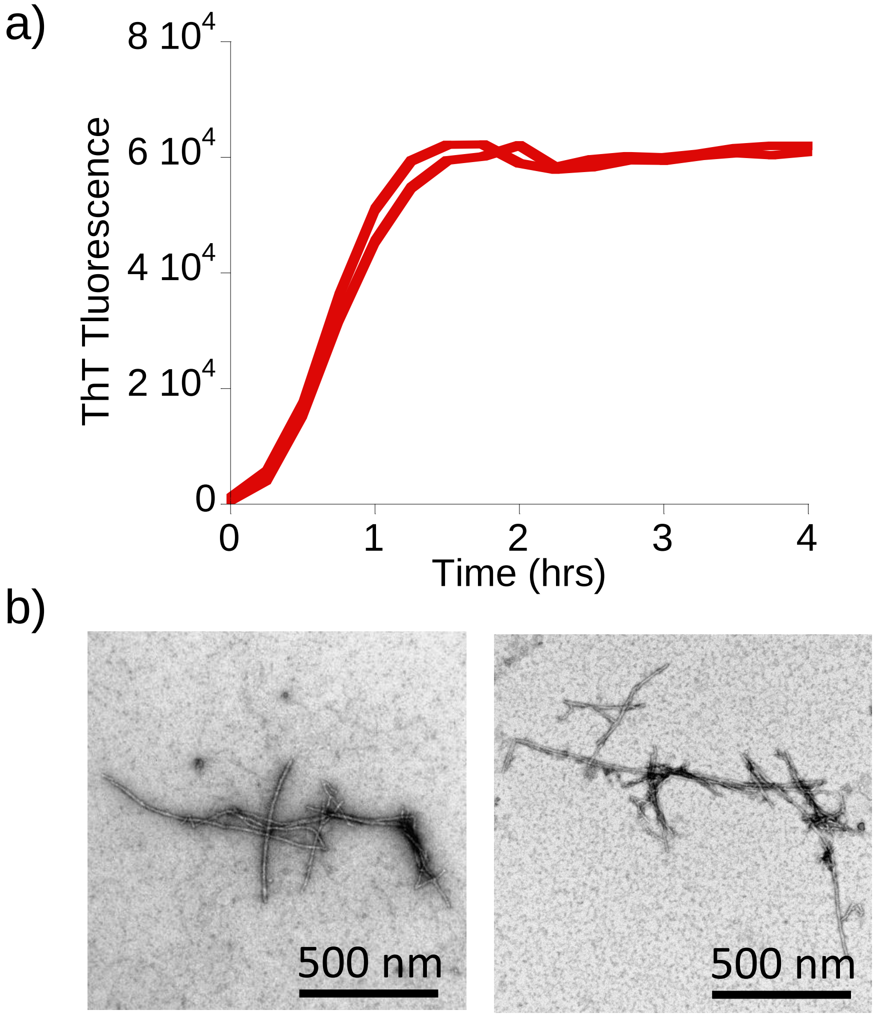

Supplemental Figure S8: **C-Amidated Aβ42** (a) Fibril growth kinetics of C-Amidated Aβ42. C-amidated Aβ42 (10 μM) was incubated with ThT (20 μM) in aqueous buffer containing NaCl (160 mM), HEPEs (30 mM), at pH 7.4. (b) TEM images of C-amidated Aβ42 fibril. Negatively stained TEM images generated from 10 μM C-amidated Aβ42 , 72 hrs incubation at pH 7.4, HEPES buffer (30 mM) and NaCl (160 mM).

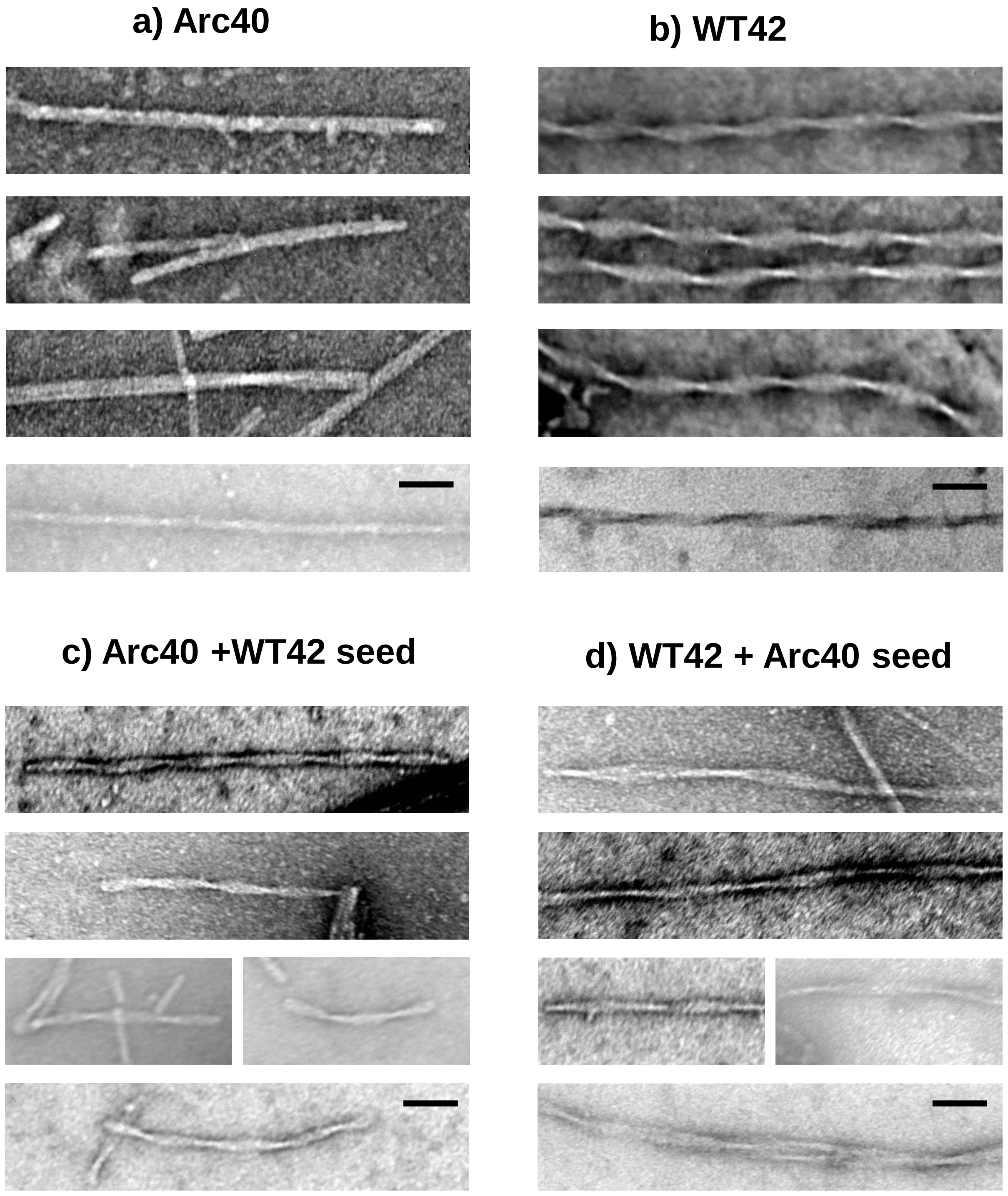

Supplemental Figure S9: TEM images of both seeded and unseeded Aβ42(WildType) and Aβ40(Arctic). a) Aβ40(Arctic); b) Aβ42(WildType); c) Aβ40(Arctic) with 10% Aβ42(WildType) fibril-seeds; d) Aβ42(WildType) with 10% Aβ40(Arctic) fibril-seeds. Aβ40(Arctic) with Aβ42(WildType) fibril-seeds added indicates cross-seeding as the Aβ42(WildType) fibril seed induces a marked twist in the otherwise untwisted Aβ40(Arctic) fibril isoform. While Aβ40(Arctic) fibril seeds cause a marked extension in the periodicity of the Aβ42(WildType) fibril twist from 80 nm to 125 nm. Scale Bar: 50 nm. (Note that Fig 7 is a simplified version of the data shown here, images are reshown here to aid direct comparison)

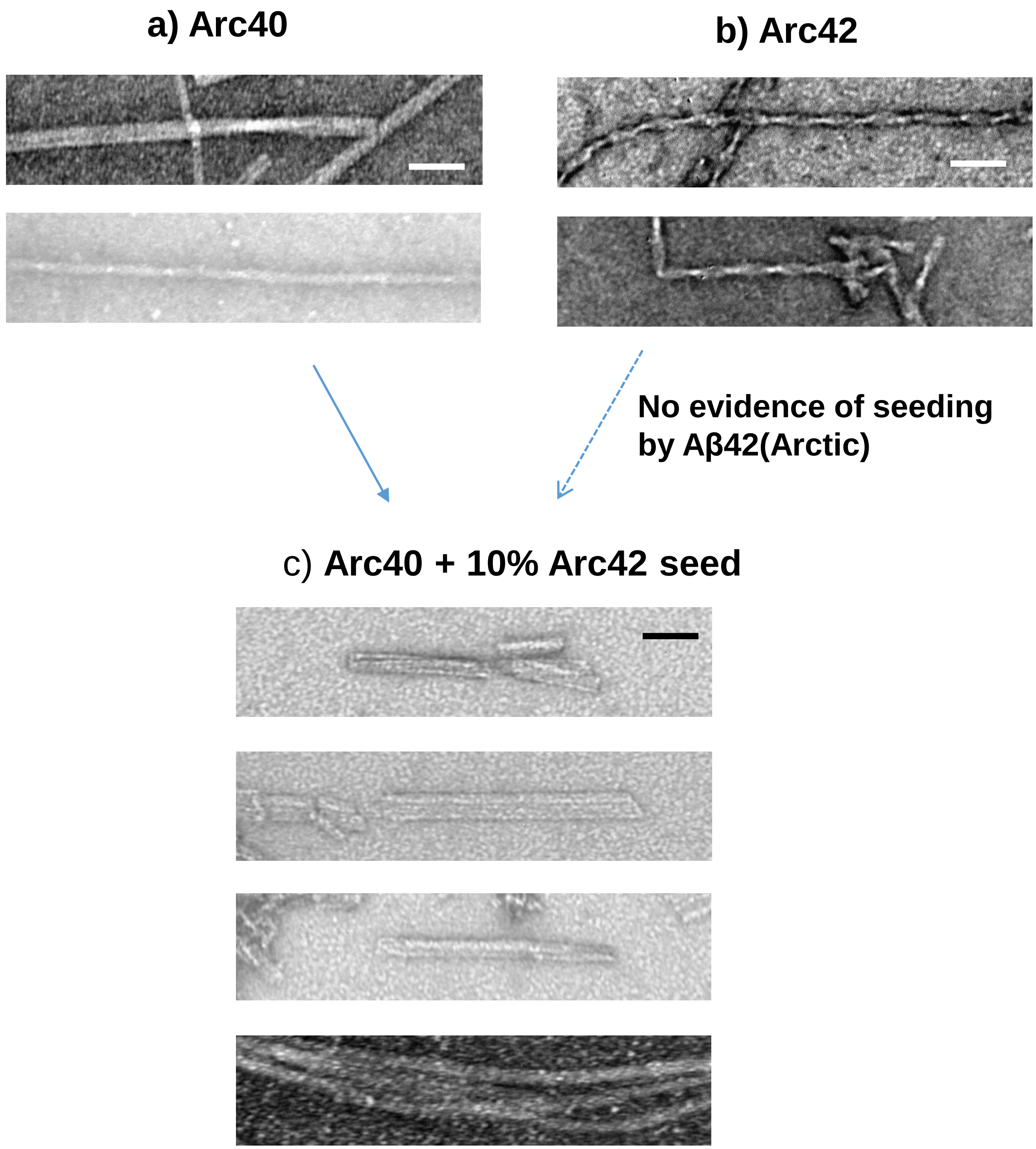

Supplemental Figure S10: TEM images of both seeded and unseeded Aβ40(Arctic). a) Aβ40(Arctic); b) Aβ42(Arctic); c) Aβ40(Arctic) with 10% Aβ42(Arctic) fibril-seeds. The morphology of Aβ40(Arctic) is unchanged by the presence of the highly twisted Aβ42(Arctic) fibril-seeds added, suggesting no cross-seeding. Scale Bar: 50 nm. (Note that the two images of unseeded Aβ40(Arctic) are reshown here to aid direct comparison with seeded fibrils and have already been shown in Fig S9a)

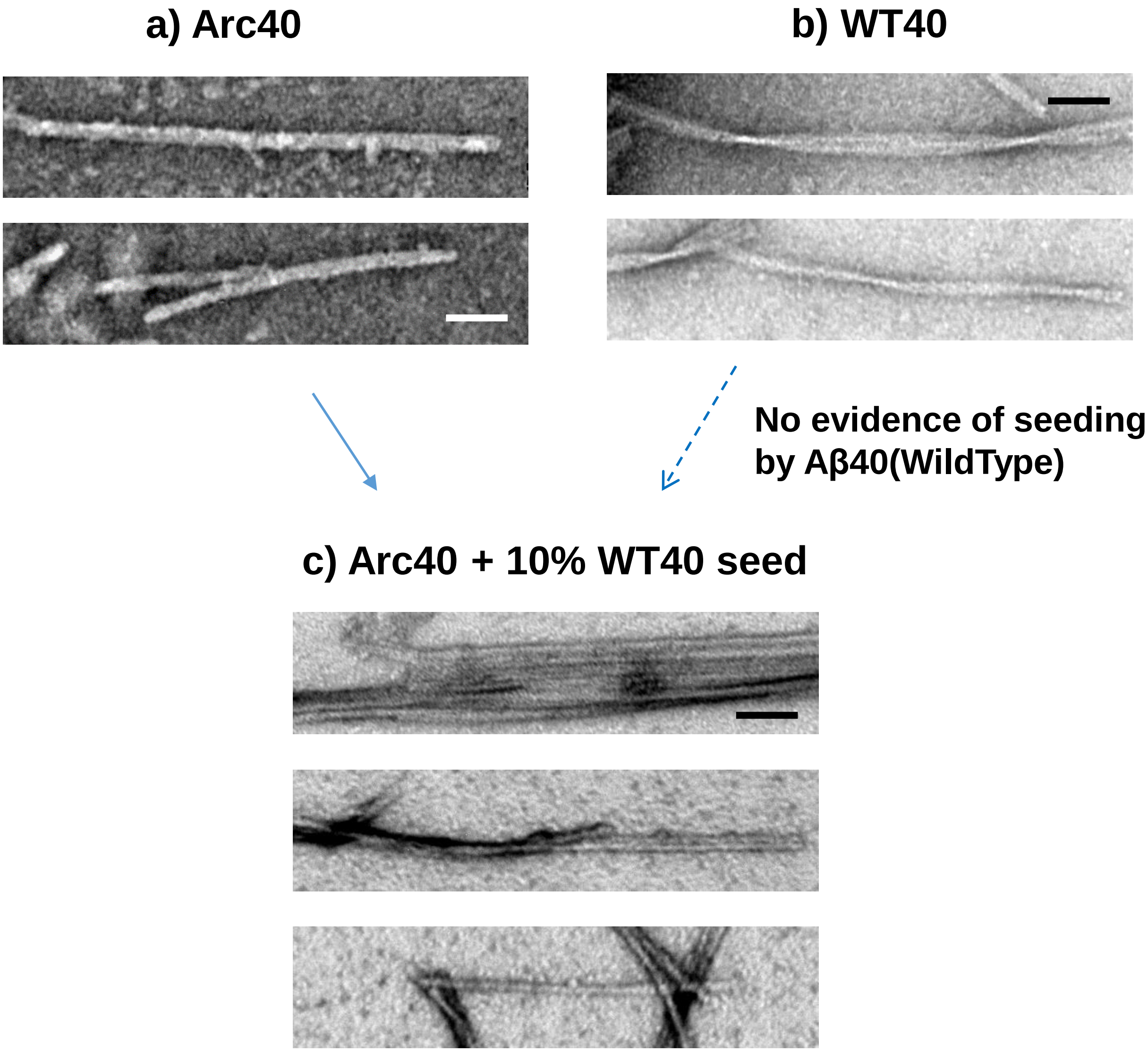

Supplemental Figure S11: TEM images of both seeded and unseeded Aβ40(Arctic), a) Aβ40(Arctic); b) Aβ40(WildType); c) Aβ40(Arctic) with 10% Aβ40(WildType) fibril-seeds. The morphology of Aβ40(Arctic) is unchanged by the more twisted presence of Aβ40(WildType) fibril-seeds added, suggesting no cross-seeding. Scale Bar: 50 nm. (Note that the two images of unseeded Aβ40(Arctic) are reshown here to aid direct comparison with seeded fibrils and have already been shown in Fig S9a)

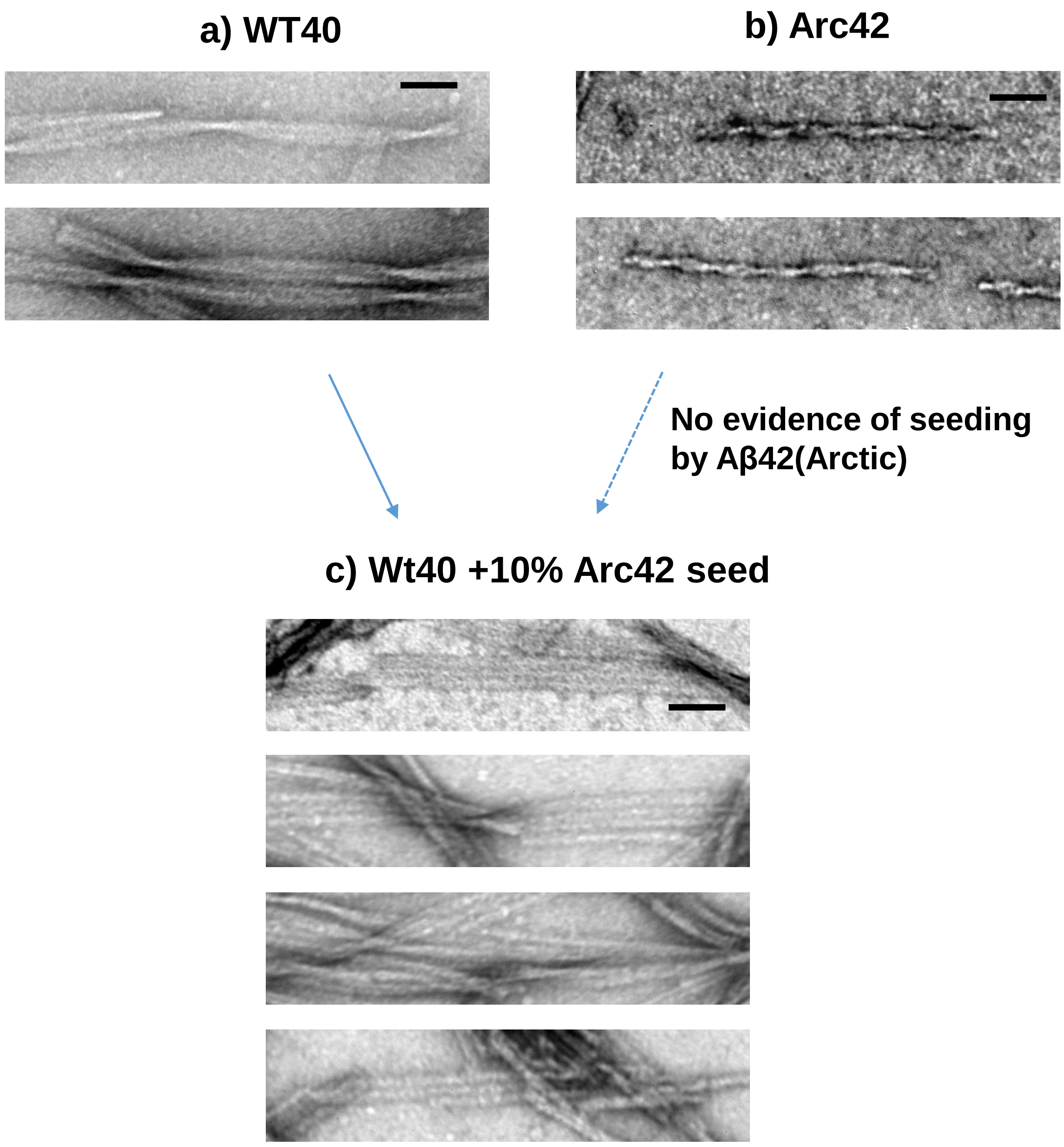

Supplemental Figure S12: TEM images of both seeded and unseeded Aβ40(WildType) a) Aβ40(WildType); b) Aβ42(Arctic); c) Aβ40(WildType) with 10% Aβ42(Arctic) fibril-seeds. The morphology of Aβ40(WildType) is unchanged by the presence of the highly twisted Aβ42(Arctic) fibril-seeds added, suggesting no cross-seeding. Scale Bar: 50 nm.
